# Supplementary material for: Tuneable near white-emissive two-dimensional covalent organic frameworks
Source: Nat Commun. 2018 Jun 13;9:2335. doi: 10.1038/s41467-018-04769-6 (PMC5997983; doi:10.1038/s41467-018-04769-6)
Supplement: Supplementary file 1 — Supplementary Information [file 41467_2018_4769_MOESM1_ESM.pdf]

Tuneable Near White-Emissive Two-Dimensional Covalent Organic Frameworks

Li *et al.*

Department of Chemistry, National University of Singapore, 3 Science Drive 3, Singapore 117543, Singapore.

Contents

Supplementary Figures **S2**

Supplementary Tables **S33**

Supplementary Methods **S55**

General Information S55

General Synthetic Procedure for Hydrazides S57

General Synthetic Procedure of Tuneable White-emissive COFs S58

General Synthetic Procedure of Model Compounds S60

General Synthetic Procedure of Multi-Component COFs S61

Supplementary Notes **62**

Supplementary Discussion **63**

Supplementary References **65**

Supplementary Figures

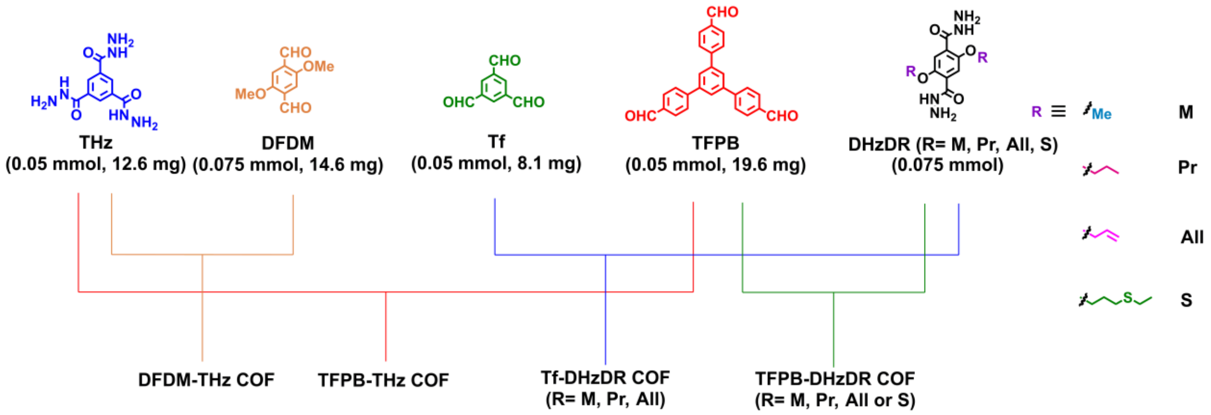

Supplementary Figure 1 | Synthesis of tuneable white-emissive COFs.

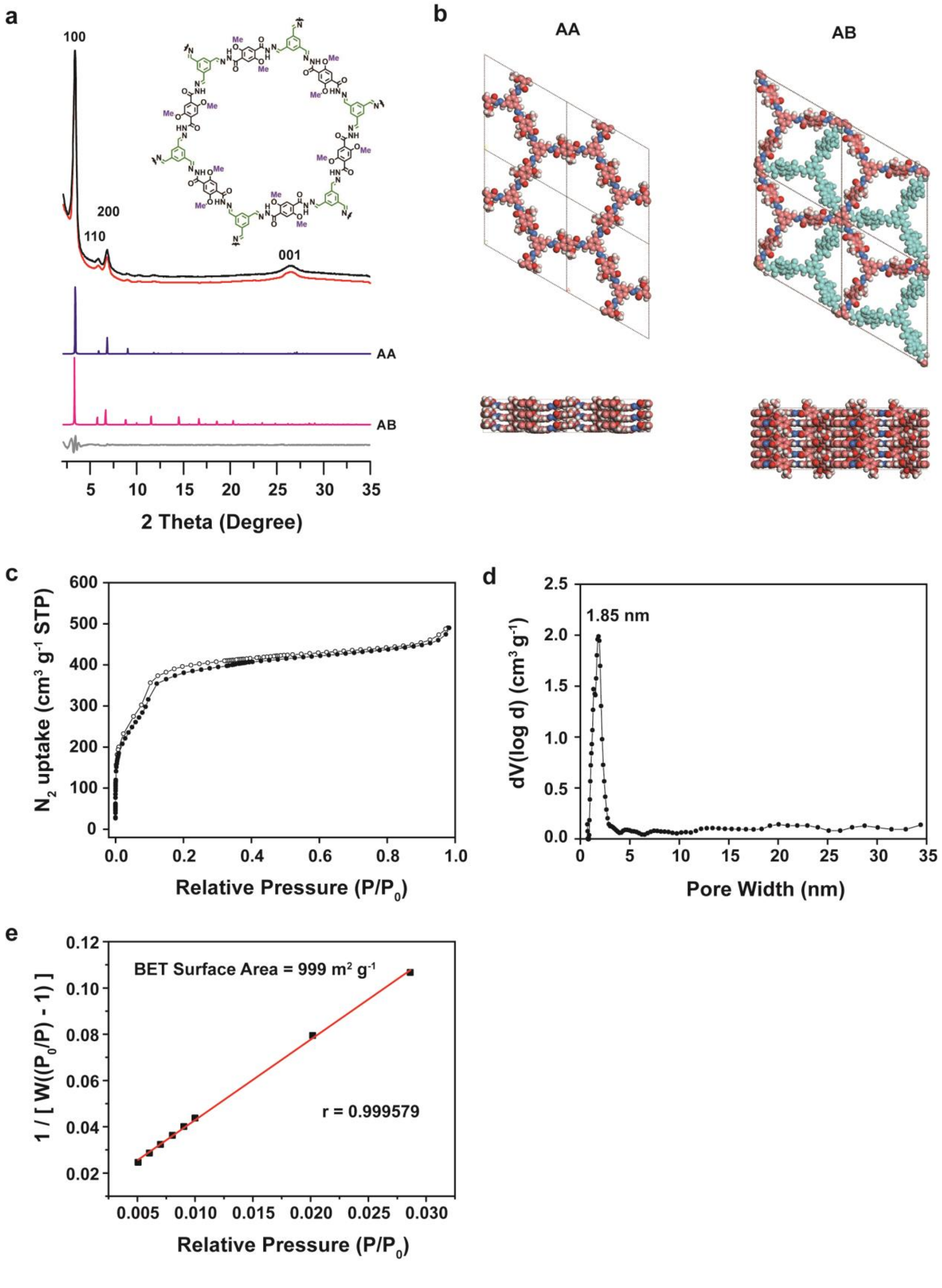

Supplementary Figure 2 | Characterizations of Tf-DHzDM COF. **a**, PXRD of Tf-DHzDM COF (experimental: black; Pawley refined: red). **b**, Simulated structure of AA stacking and AB stacking. **c**, N_2 sorption isotherm of Tf-DHzDM COF. **d**, Pore distribution of Tf-DHzDM COF. **e**, Multipoint BET plot of Tf-DHzDM.

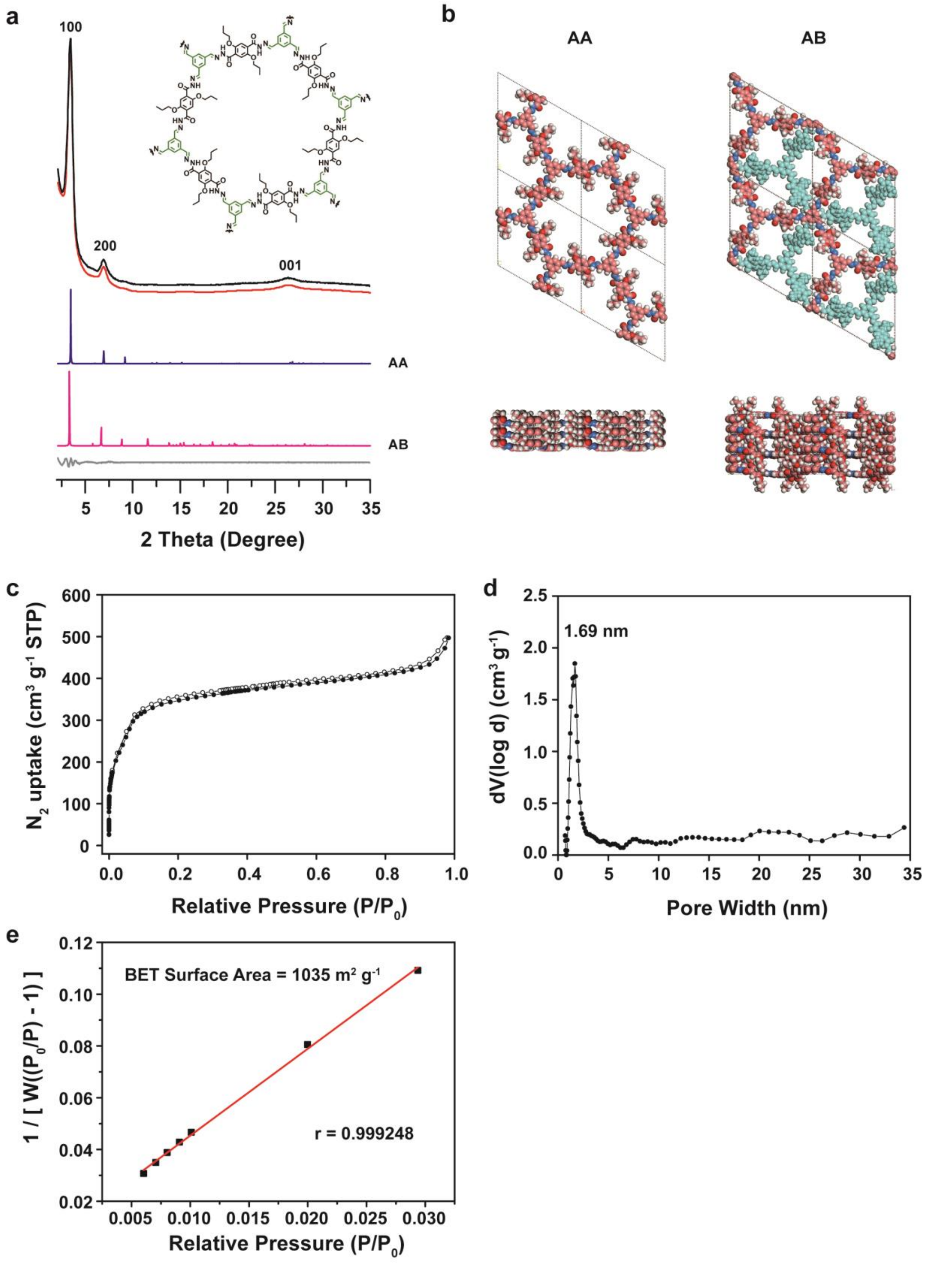

Supplementary Figure 3 | Characterizations of Tf-DHzDPr COF. a, PXRD of Tf-DHzDPr COF (experimental: black; Pawley refined: red). **b**, Simulated structure of AA stacking and AB stacking. **c**, N_2 sorption isotherm of Tf-DHzDPr COF. **d**, Pore distribution of Tf-DHzDPr COF. **e**, Multipoint BET plot of Tf-DHzDPr.

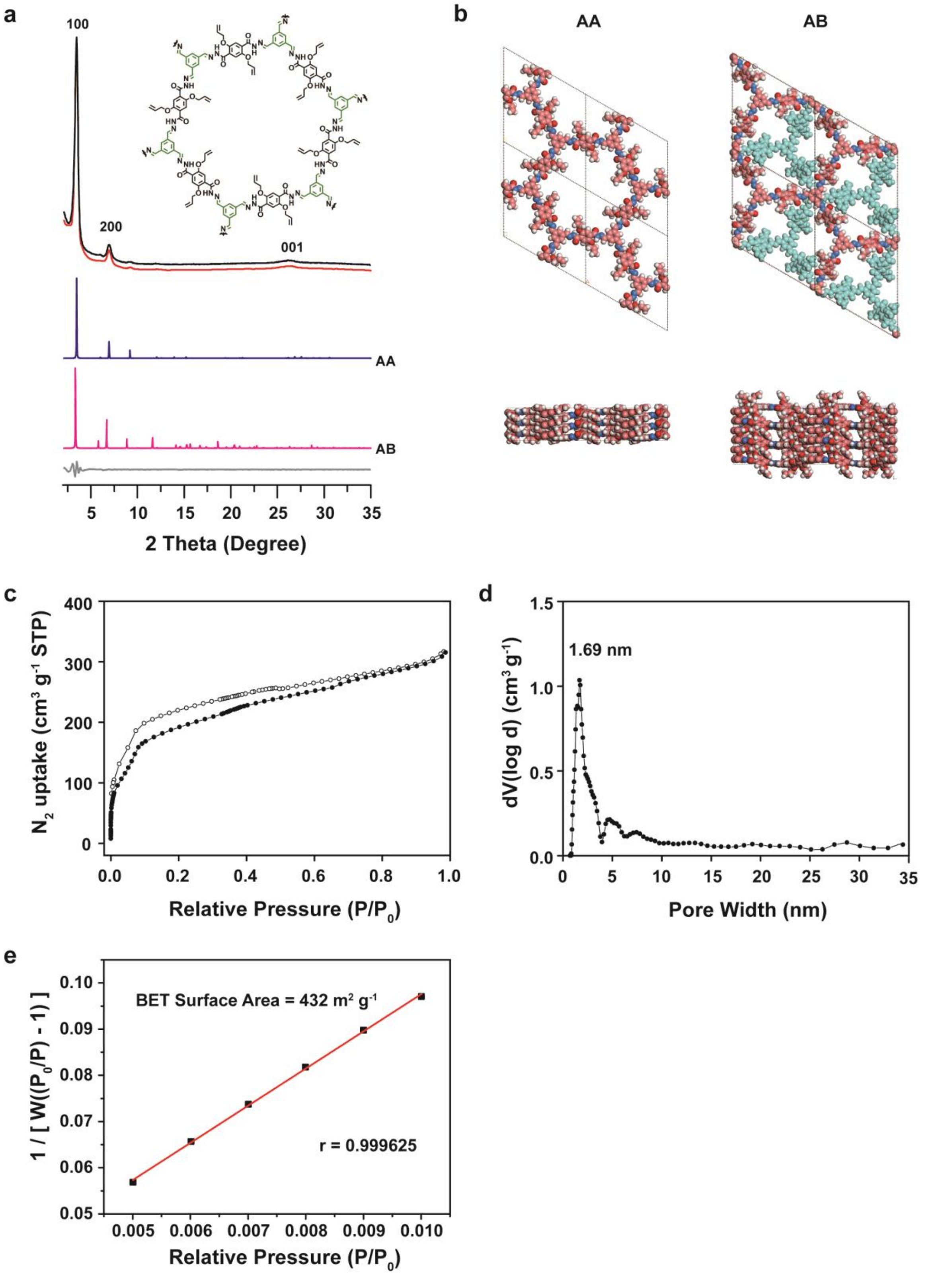

Supplementary Figure 4 | Characterizations of Tf-DHzDAll COF. a, PXRD of Tf-DHzDAll COF (experimental: black; Pawley refined: red). **b**, Simulated structure of AA stacking and AB stacking. **c**, N_2 sorption isotherm of Tf-DHzDAll COF. **d**, Pore distribution of Tf-DHzDAll COF. **e**, Multipoint BET plot of Tf-DHzDAll.

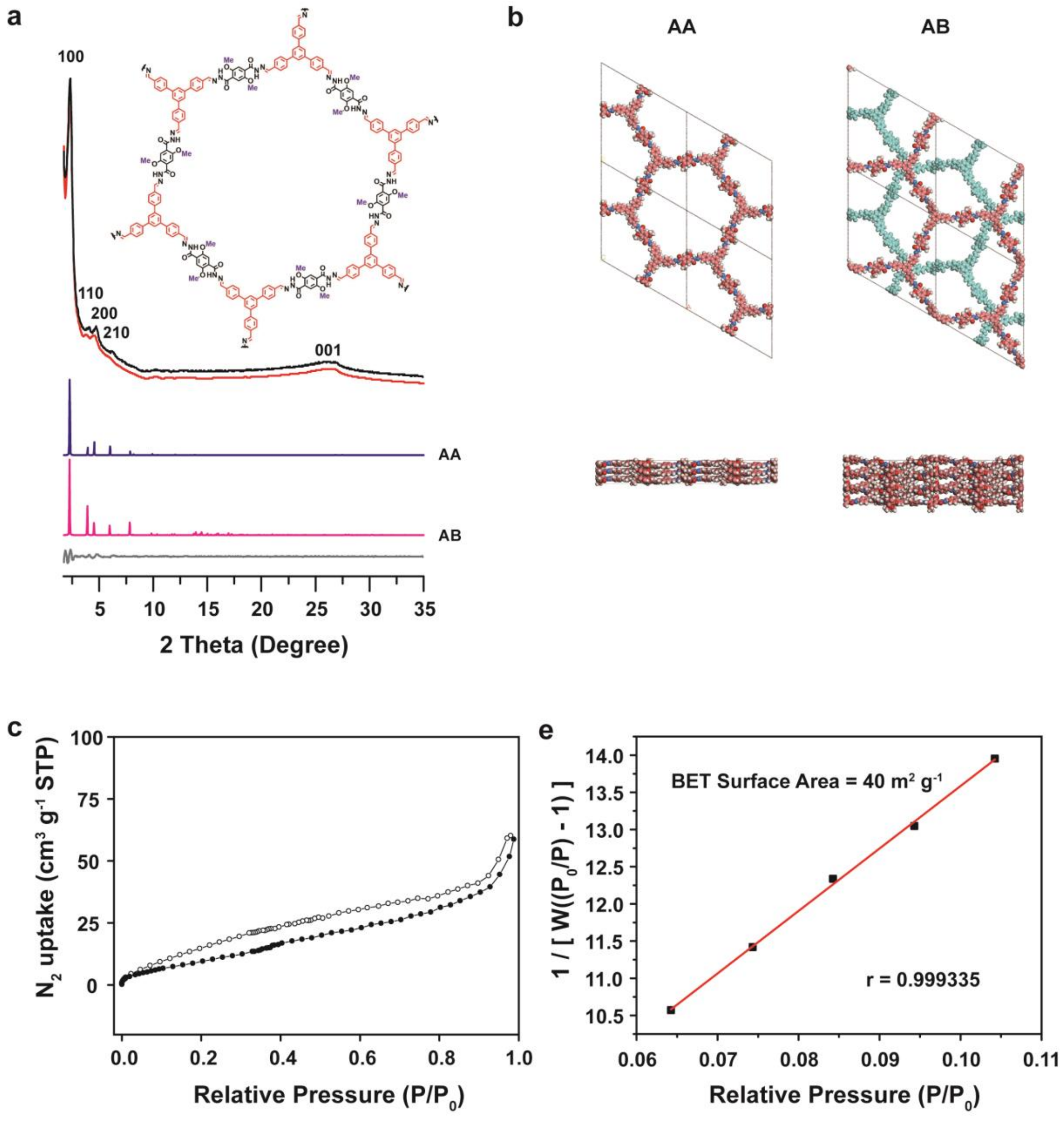

Supplementary Figure 5 | Characterizations of TFPB-DHzDM COF. a, PXRD of TFPB-DHzDM COF (experimental: black; Pawley refined: red). **b**, Simulated structure of AA stacking and AB stacking. **c**, N_2 sorption isotherm of TFPB-DHzDM COF. **d**, Multipoint BET plot of TFPB-DHzDM.

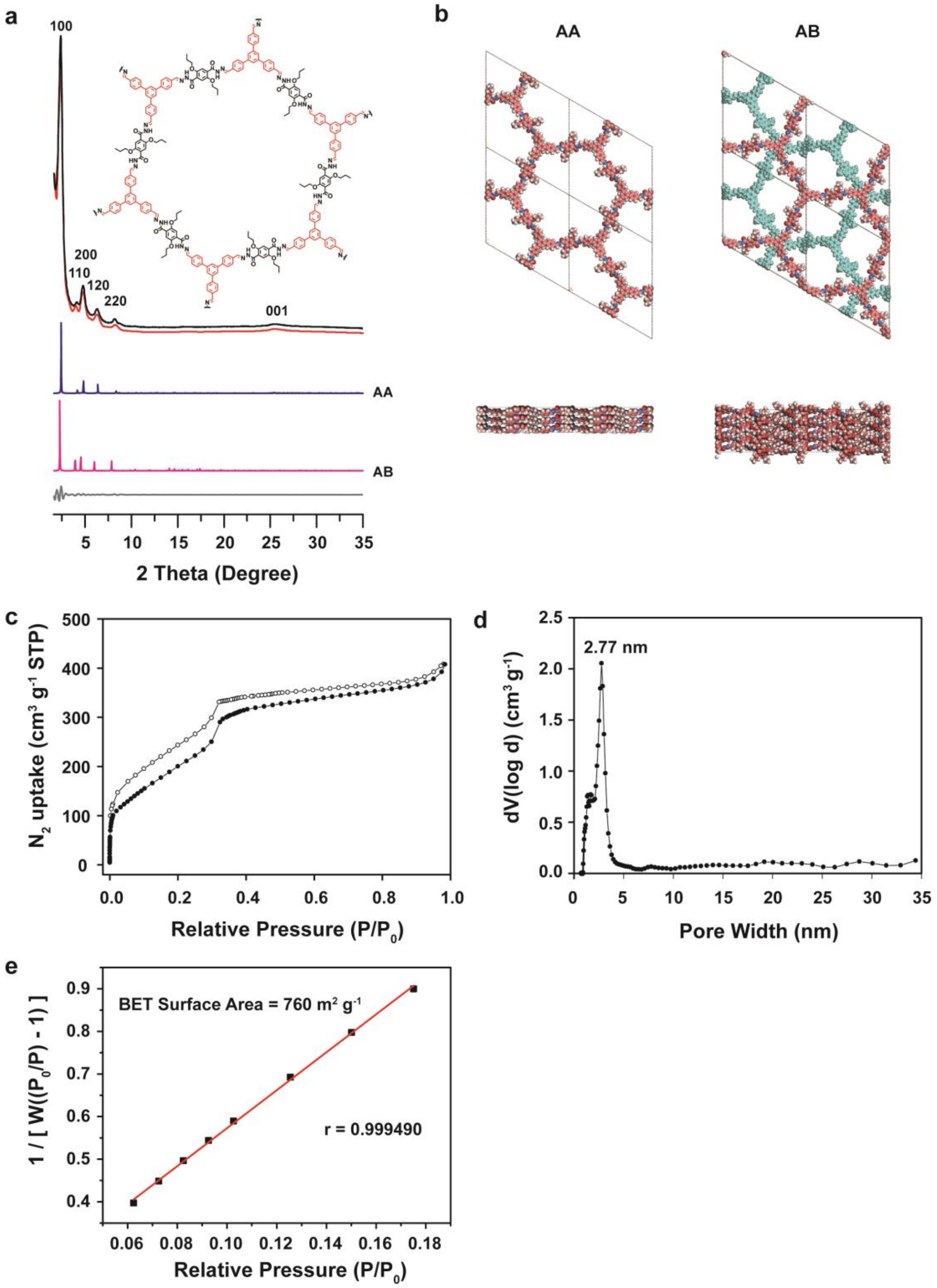

Supplementary Figure 6 | Characterizations of TFPB-DHzDPr COF. a, PXRD of TFPB-DHzDPr COF (experimental: black; Pawley refined: red). **b**, Simulated structure of AA stacking and AB stacking. **c**, N_2 sorption isotherm of TFPB-DHzDPr COF. **d**, Pore distribution of TFPB-DHzDPr COF. **e**, Multipoint BET plot of TFPB-DHzDPr.

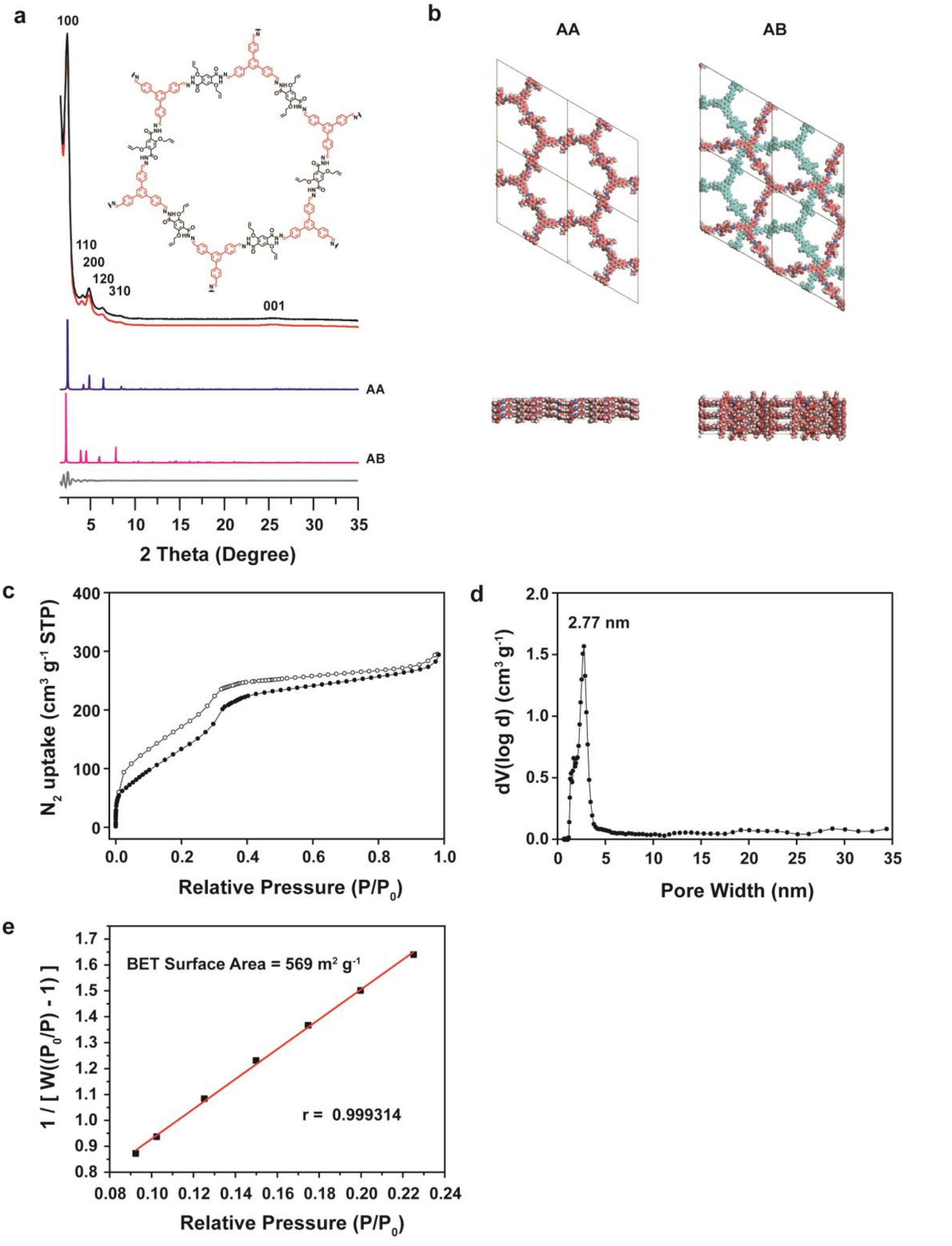

Supplementary Figure 7 | Characterizations of TFPB-DHzDAll COF. a, PXRD of TFPB-DHzDAll COF (experimental: black; Pawley refined: red). **b**, Simulated structure of AA stacking and AB stacking. **c**, N_2 sorption isotherm of TFPB-DHzDAll COF. **d**, Pore distribution of TFPB-DHzDAll COF. **e**, Multipoint BET plot of TFPB-DHzDAll.

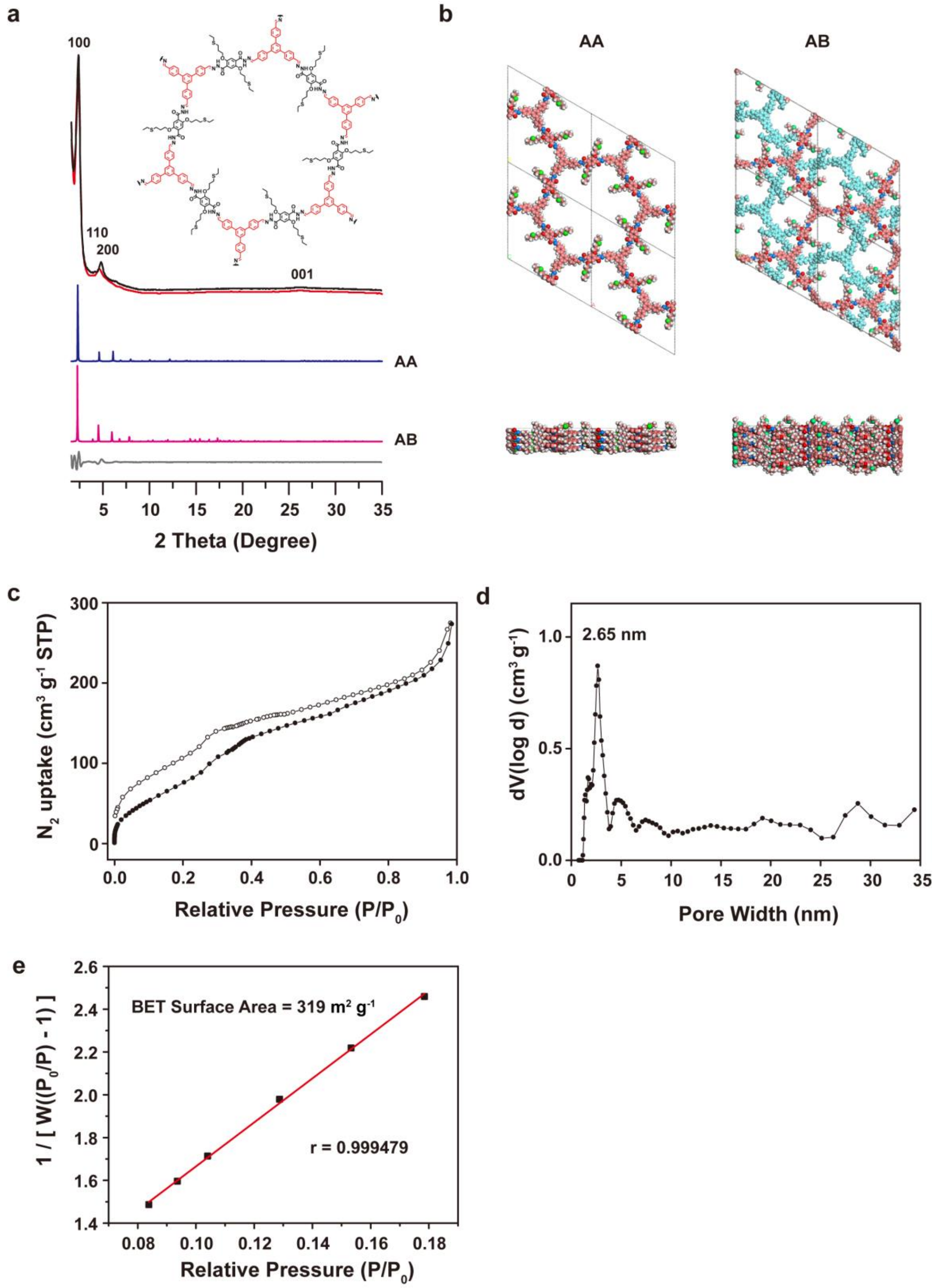

Supplementary Figure 8 | Characterizations of TFPB-DHzDS COF. a, PXRD of TFPB-DHzDS COF (experimental: black; Pawley refined: red). **b**, Simulated structure of AA stacking and AB stacking. **c**, N_2 sorption isotherm of TFPB-DHzDS COF. **d**, Pore distribution of TFPB-DHzDS COF. **e**, Multipoint BET plot of TFPB-DHzDS.

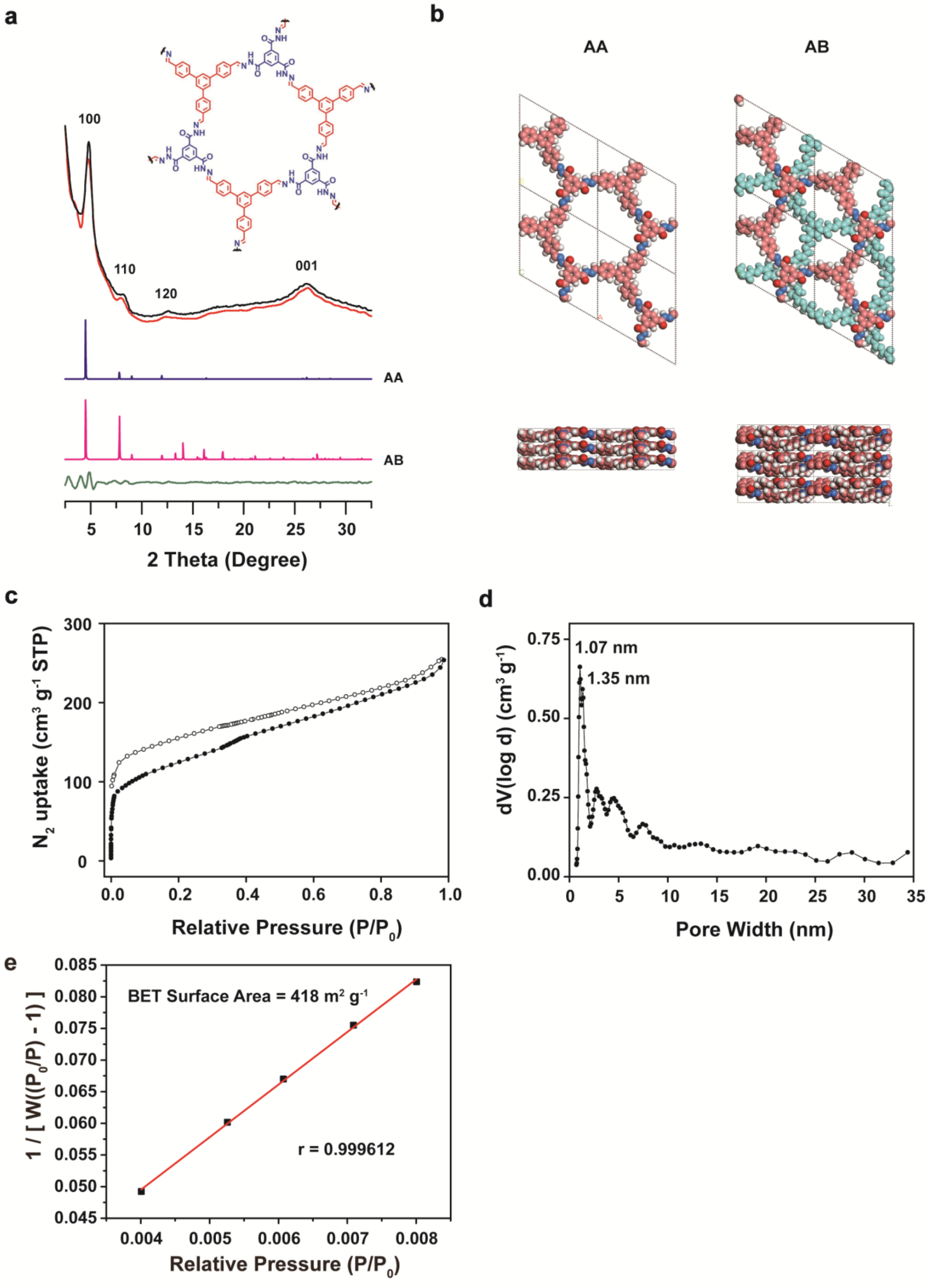

Supplementary Figure 9 | Characterizations of TFPB-THz COF. a, PXRD of TFPB-THz COF (experimental: black; Pawley refined: red). **b**, Simulated structure of AA stacking and AB stacking. **c**, N_2 sorption isotherm of TFPB-THz COF. **d**, Pore distribution of TFPB-THz COF. **e**, Multipoint BET plot of TFPB-THz.

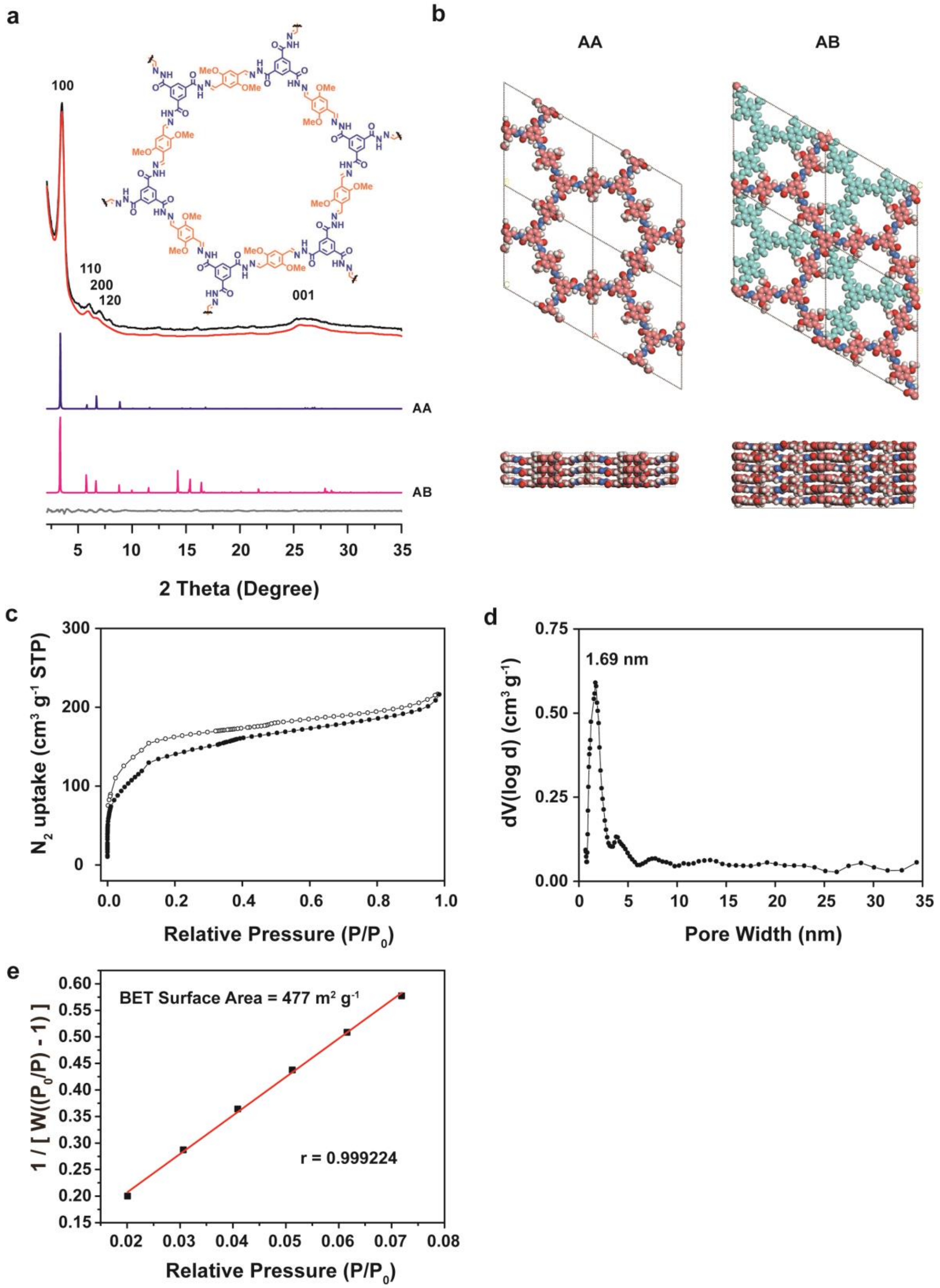

Supplementary Figure 10 | Characterizations of DFDM-THz COF. **a**, PXRD of DFDM-THz COF (experimental: black; Pawley refined: red). **b**, Simulated structure of AA stacking and AB stacking. **c**, N_2 sorption isotherm of DFDM-THz COF. **d**, Pore distribution of DFDM-THz COF.

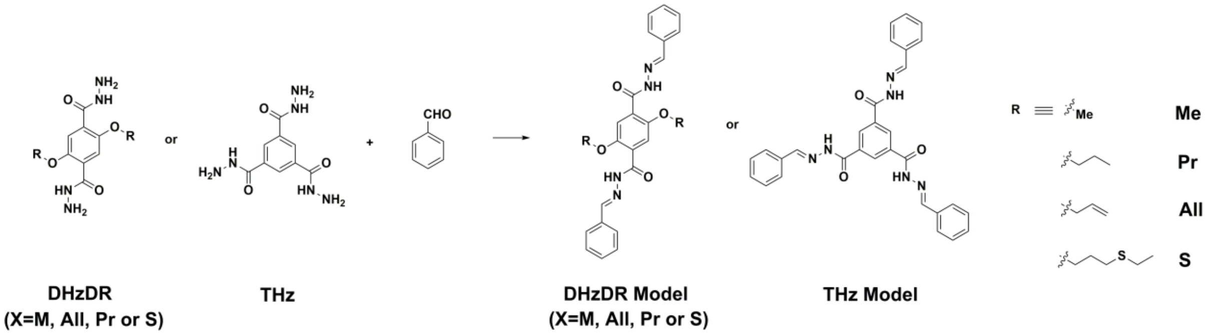

Supplementary Figure 11 | Synthesis of model compounds.

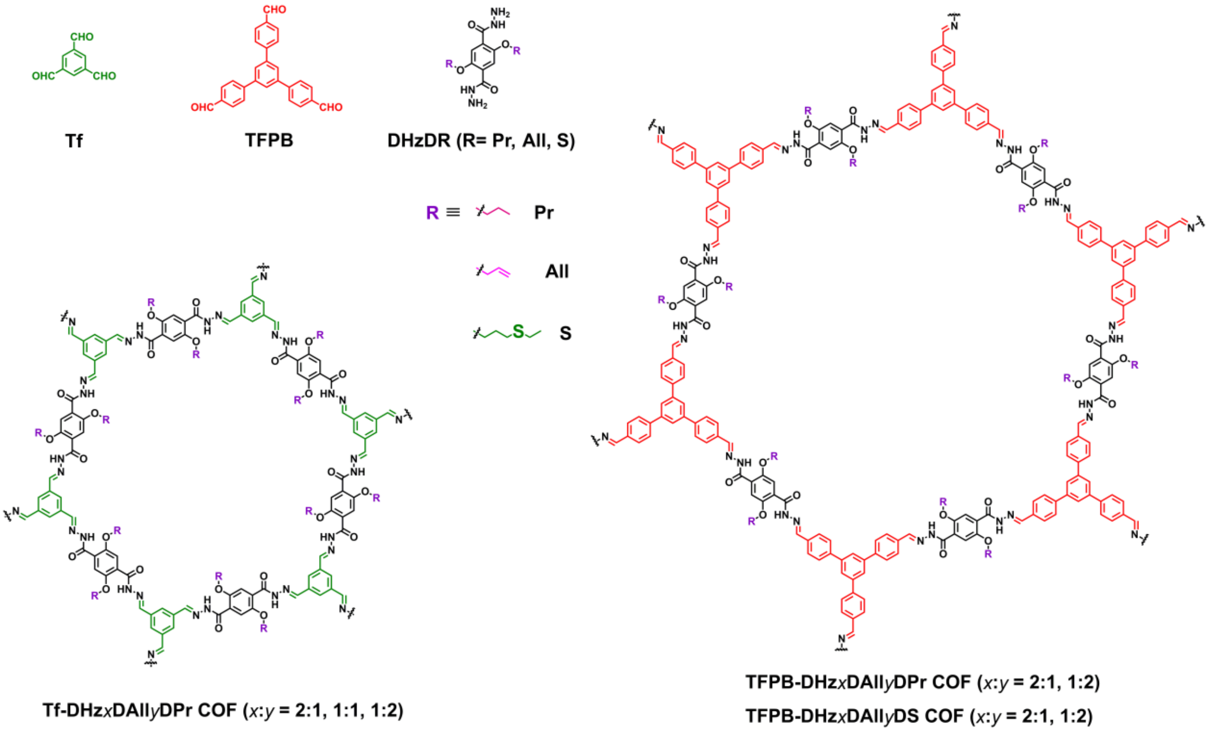

Supplementary Figure 12 | Synthesis of multi-component COFs.

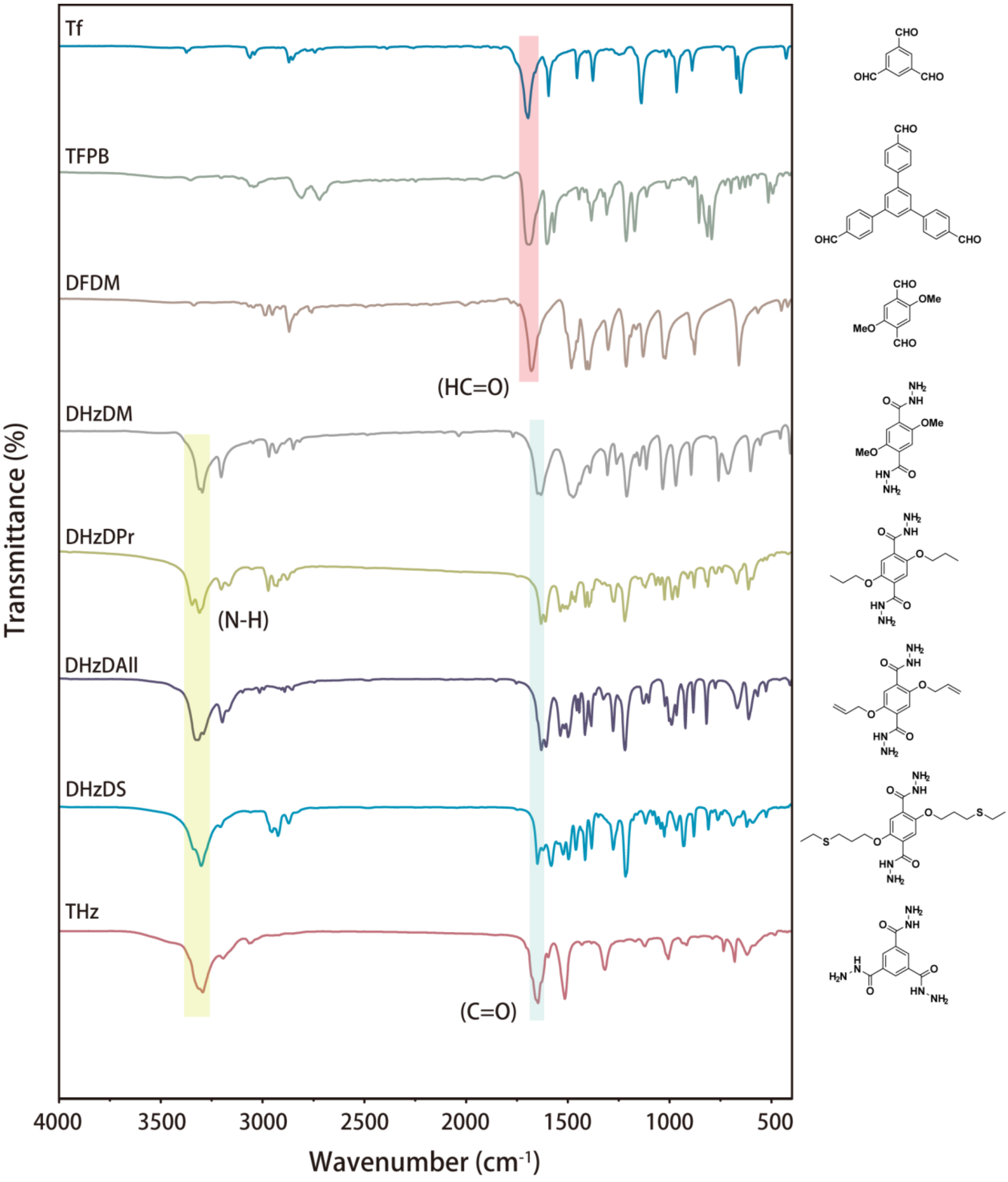

Supplementary Figure 13 | FTIR spectra of COF building units.

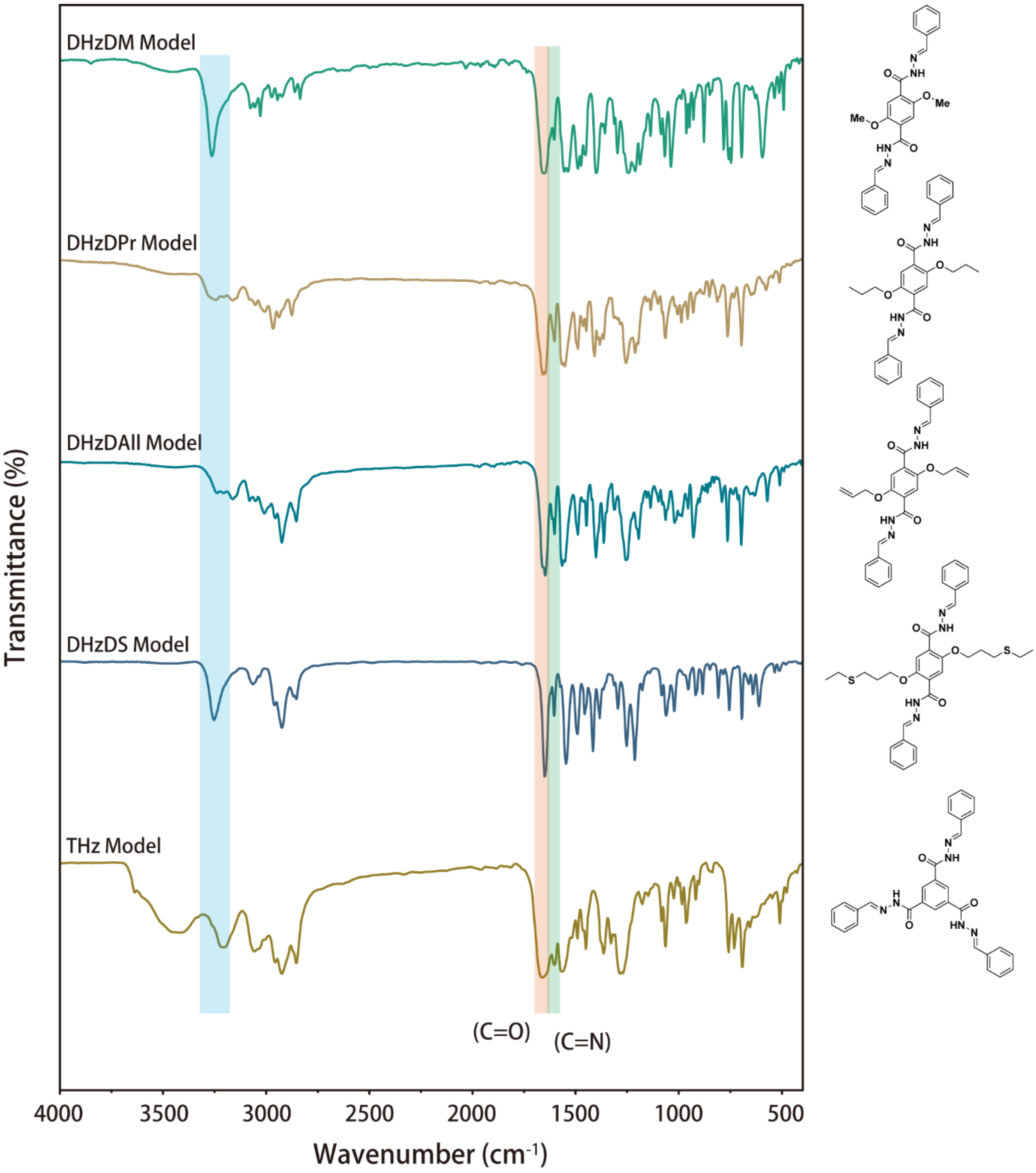

Supplementary Figure 14 | FTIR spectra of model compounds.

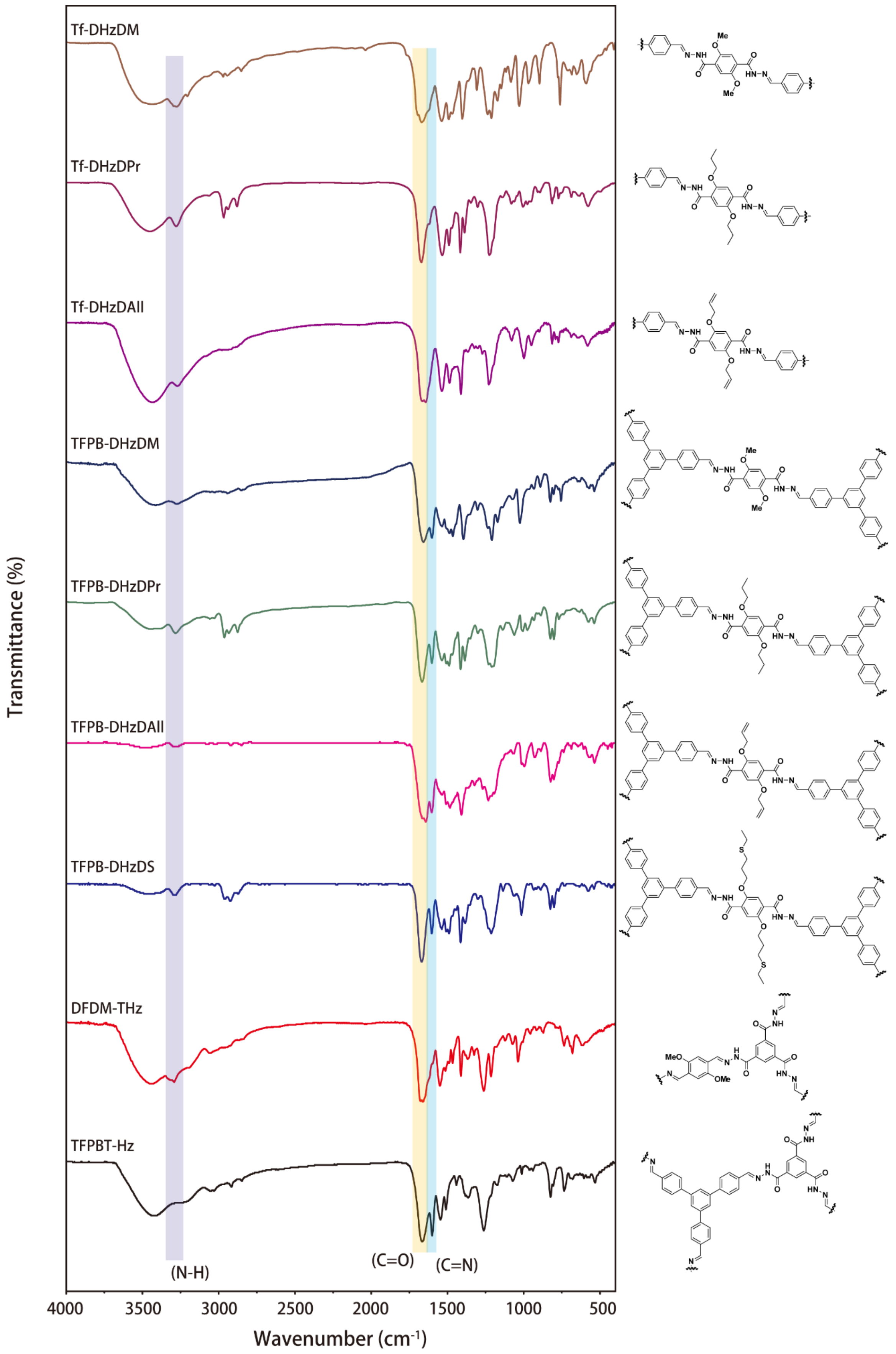

Supplementary Figure 15 | FTIR spectra of COFs.

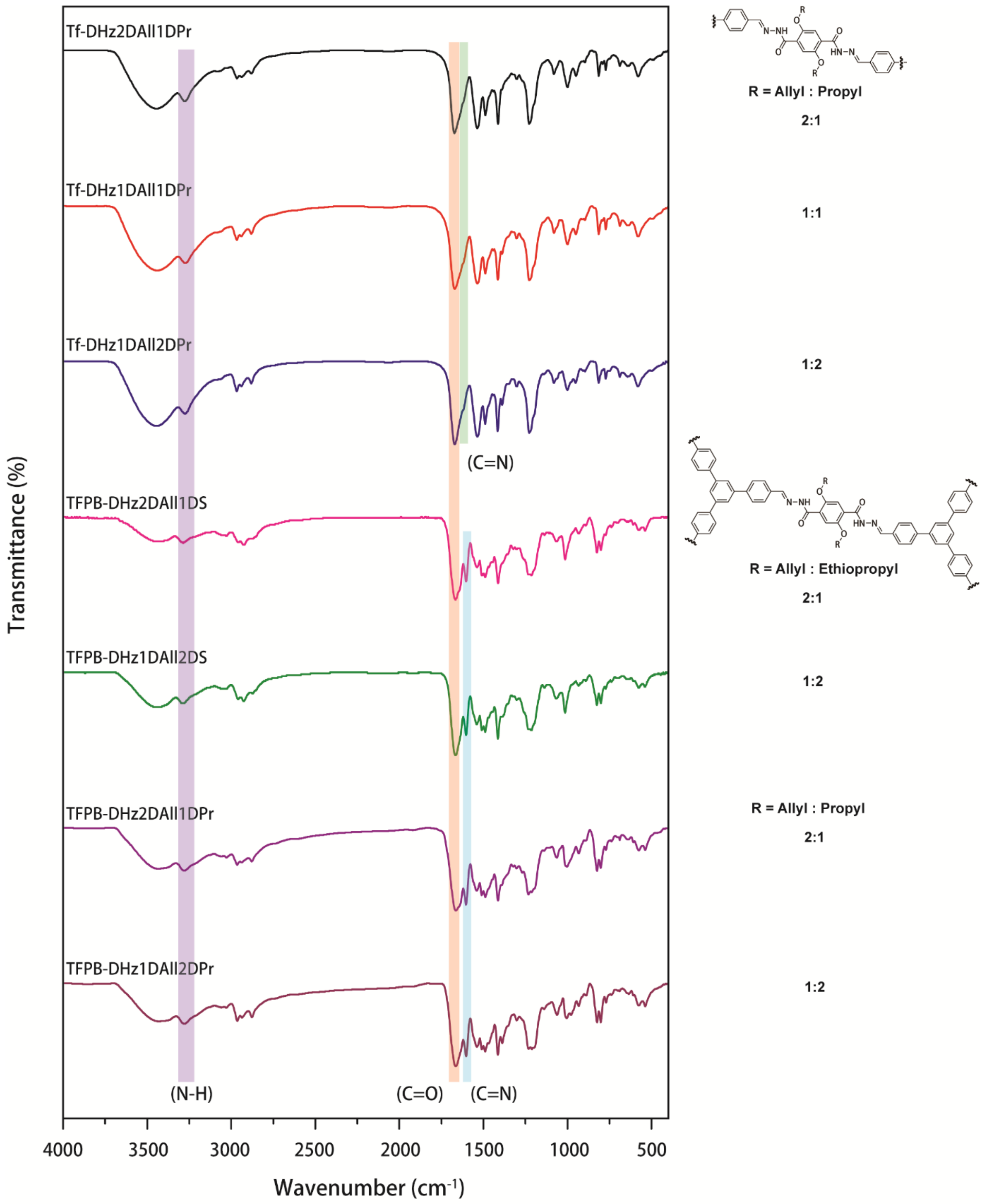

Supplementary Figure 16 | FTIR spectra of multi-component COFs.

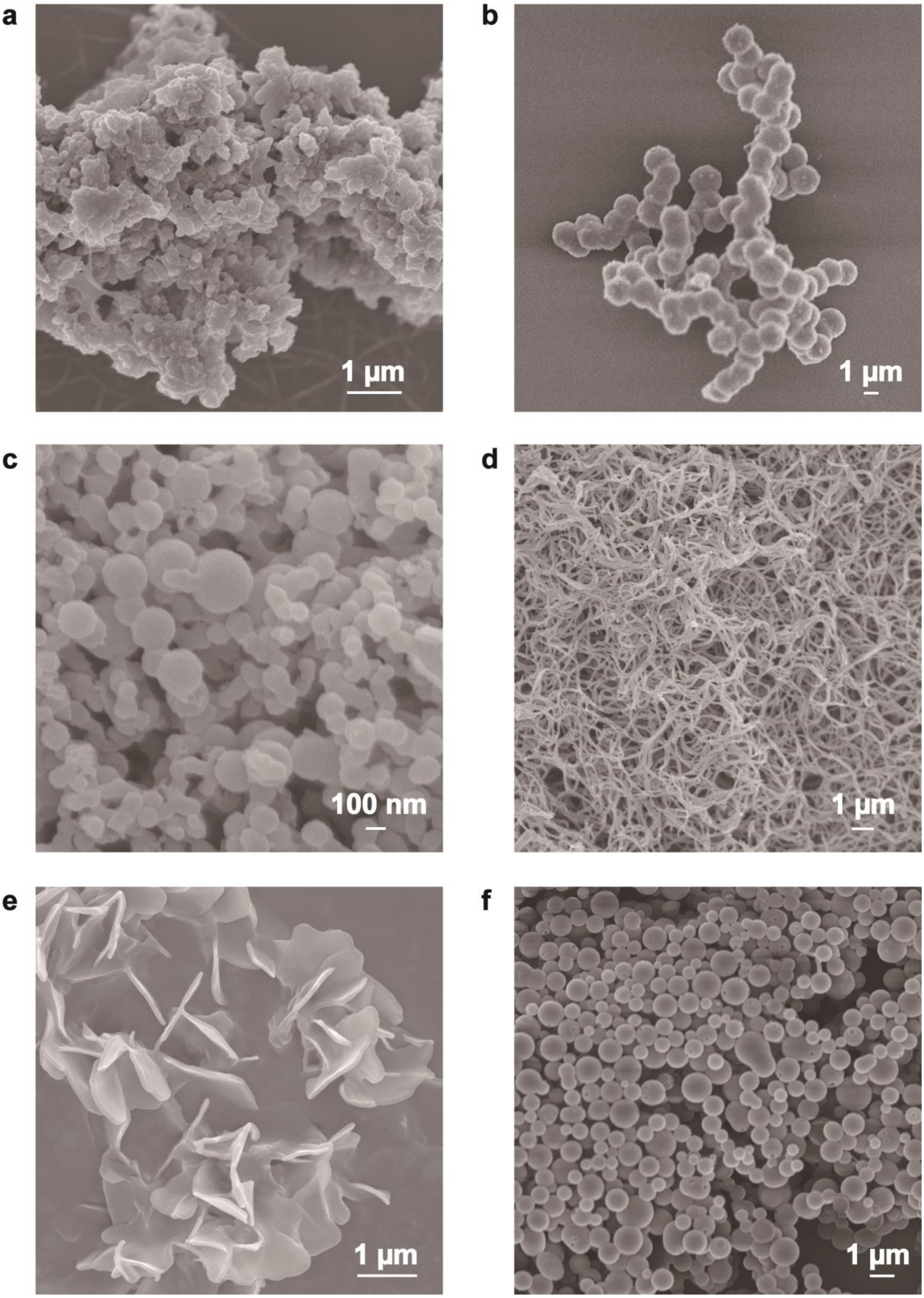

Supplementary Figure 17 | SEM images of **a**, Tf-DHzDM COF, **b**, TFPB-DHzDM COF, **c**, Tf-DHzDPr COF, **d**, TFPB-DHzDPr COF, **e**, Tf-DHzDAll COF, and **f**, TFPB-DHzDAll COF.

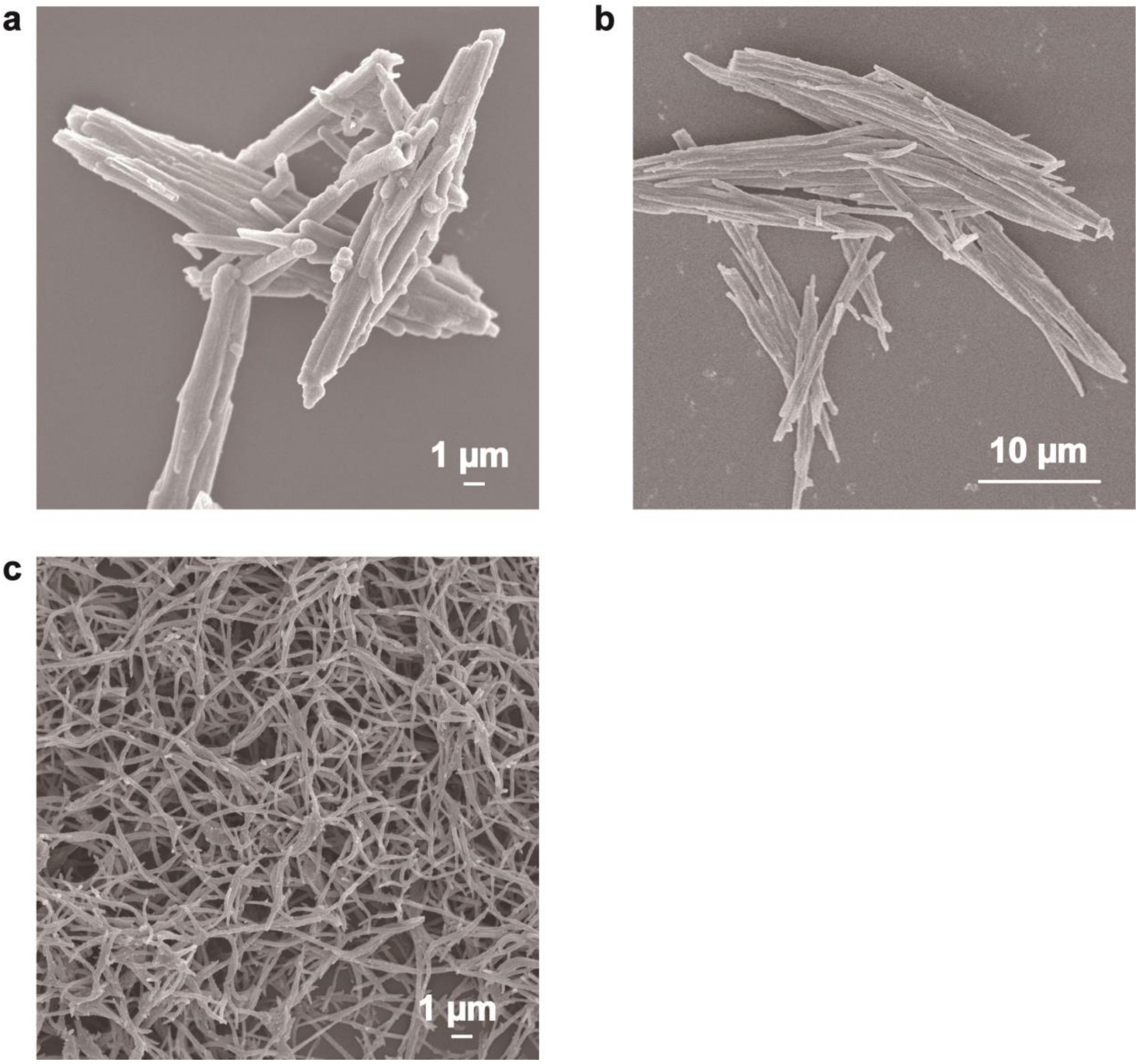

Supplementary Figure 18 | SEM images of **a**, TFPB-THz COF, **b**, DFDM-THz COF, and **c**, TFPB-DHzDS COF.

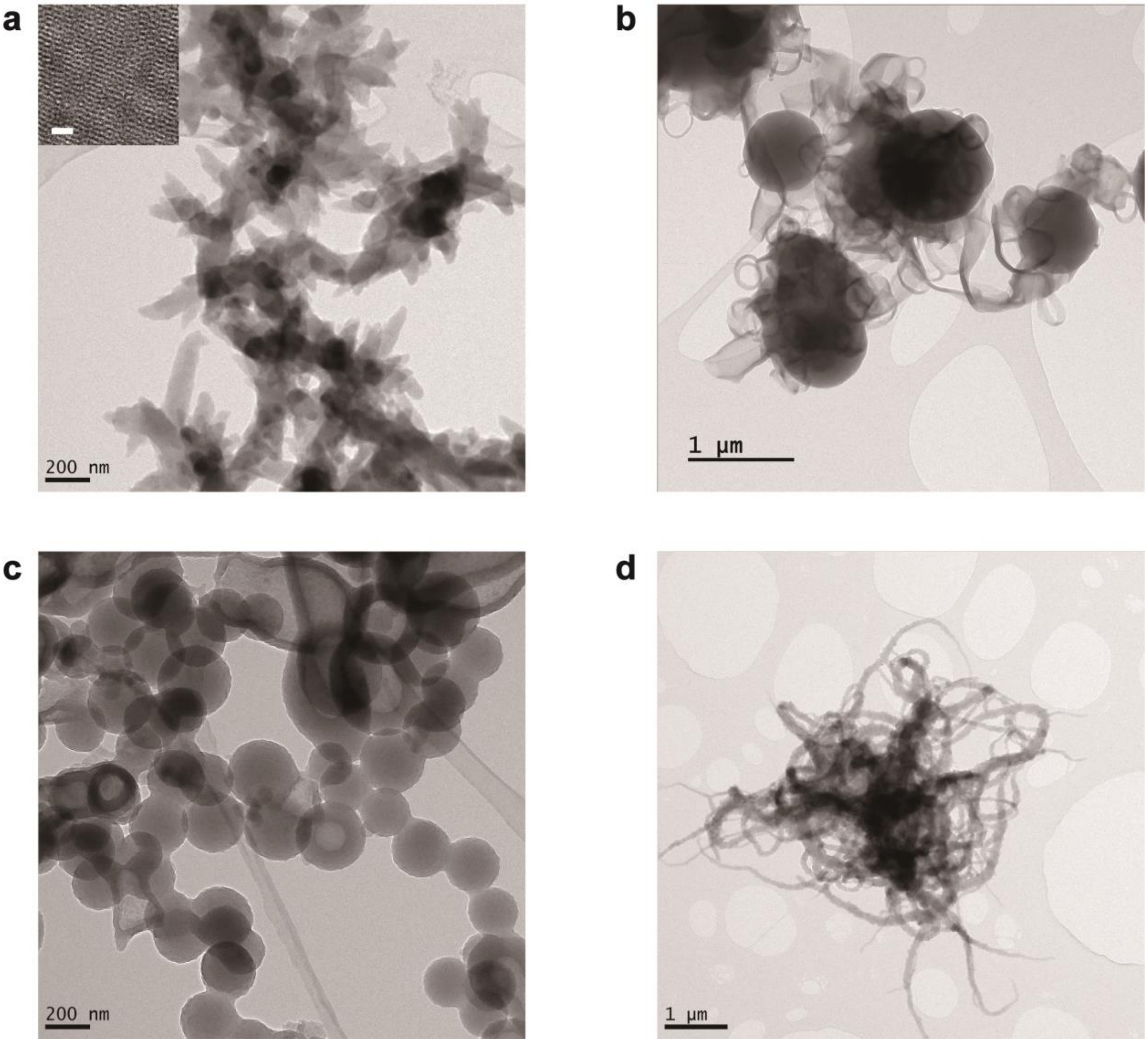

Supplementary Figure 19 | TEM images of **a**, Tf-DHzDAll COF (inlet 1 nm), **b**, TFPB-DHzDAll COF, **c**, Tf-DHzDPr COF, and **d**, TFPB-DHzDPr COF.

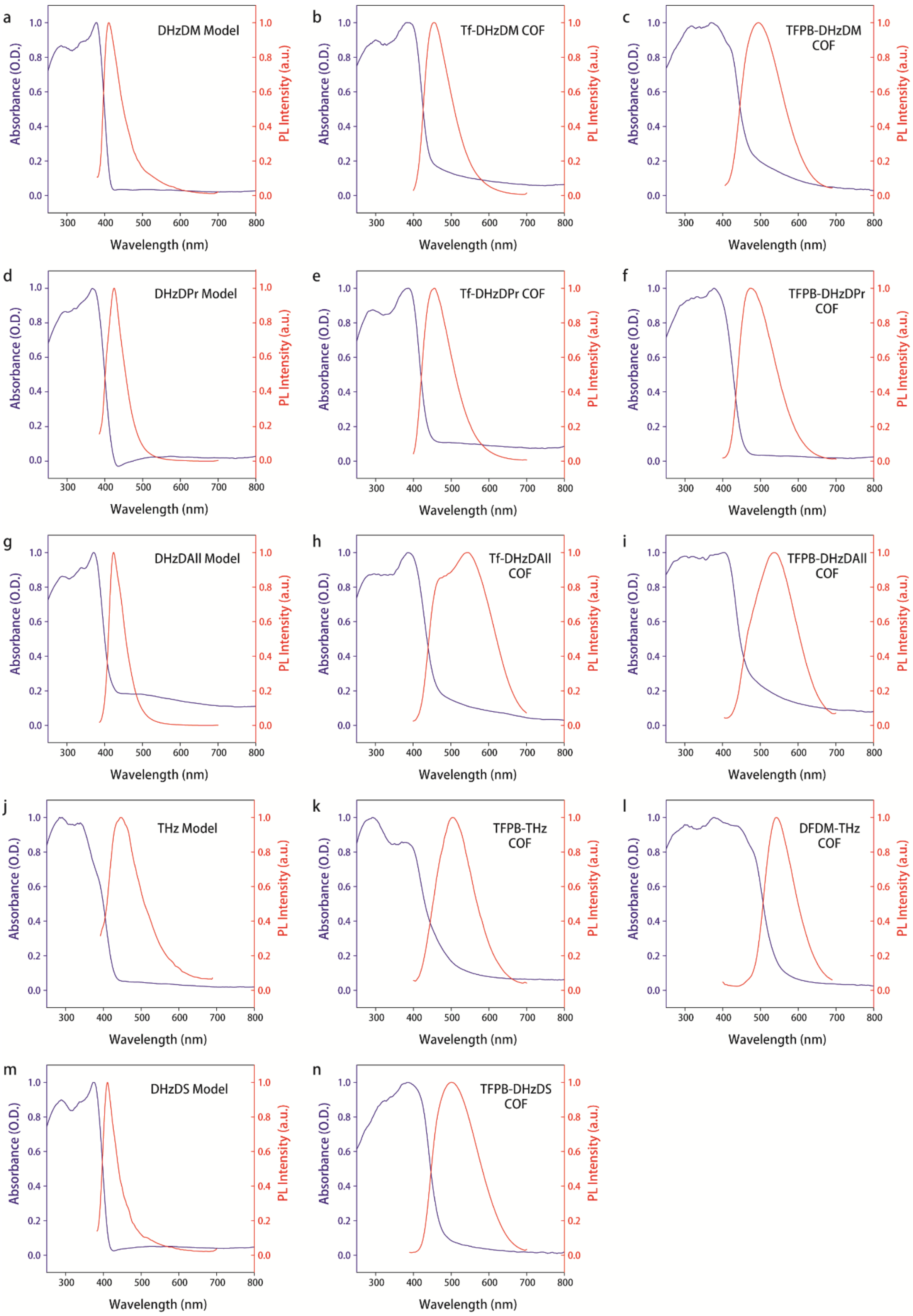

Supplementary Figure 20 | Absorption and emission spectra of various COFs and model compounds.

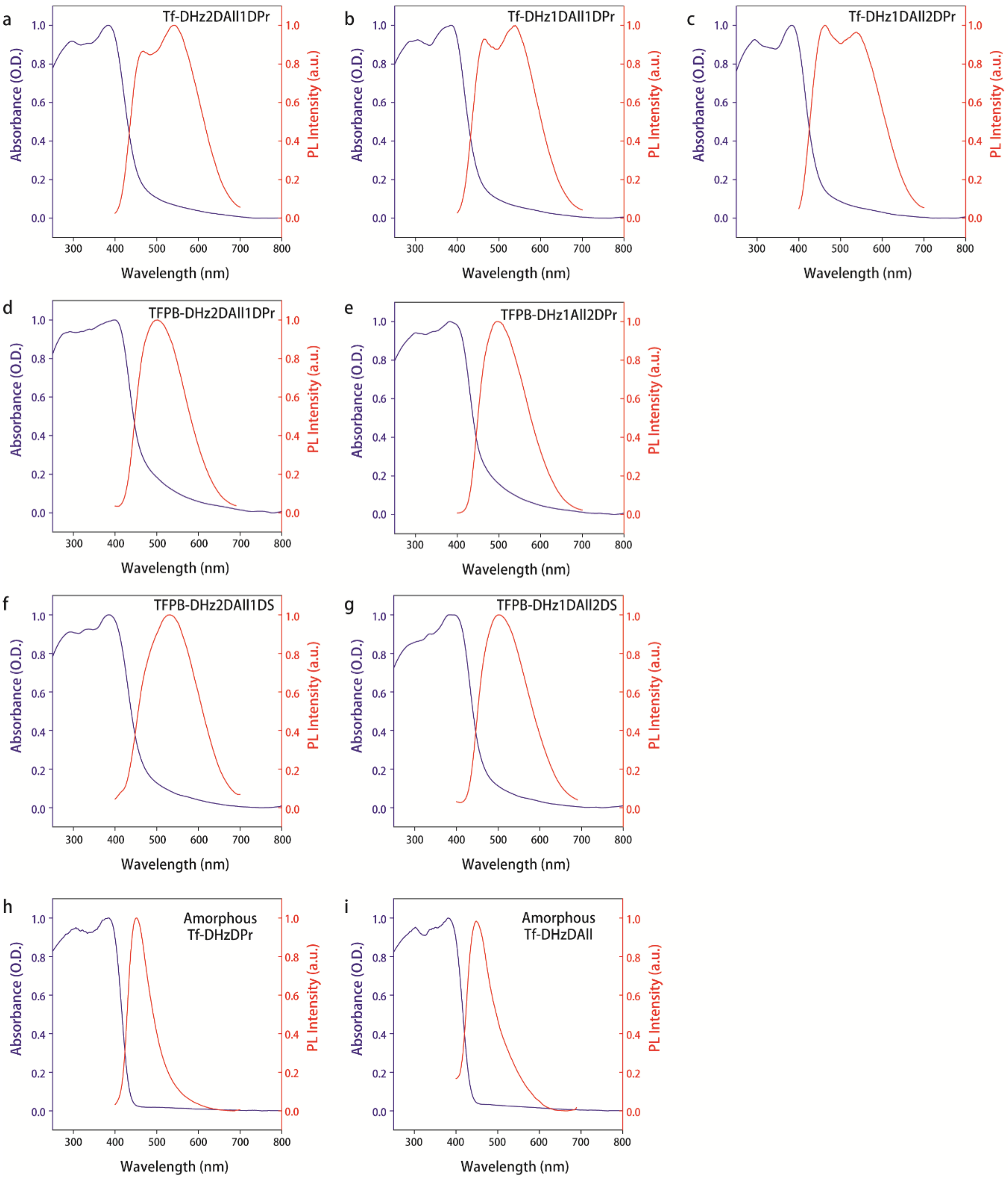

Supplementary Figure 21 | Absorption and emission spectra of multi-component COFs and amorphous Tf-DHzDPr and Tf-DHzDAll.

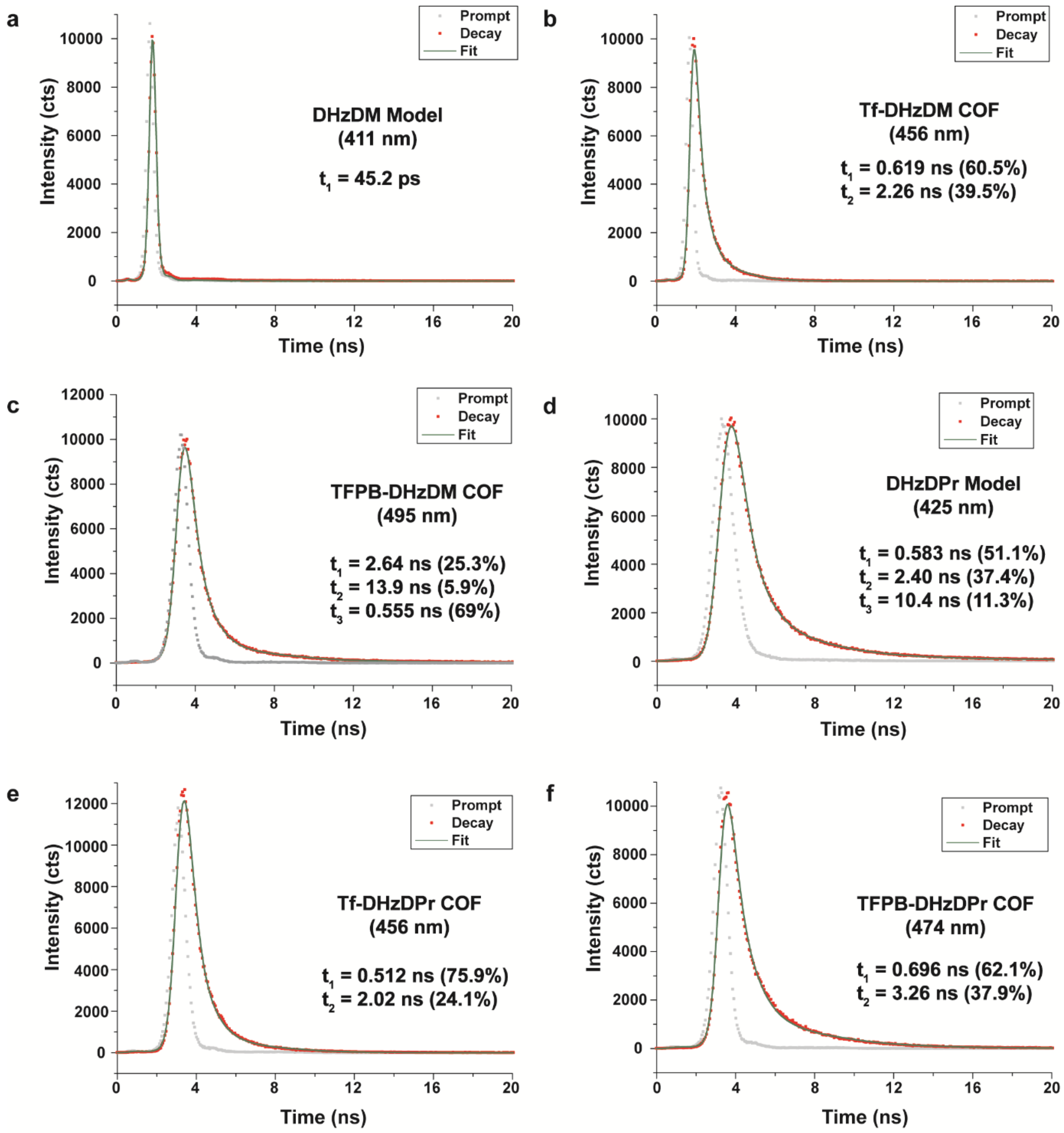

Supplementary Figure 22 | PL lifetime of various COFs and model compounds.

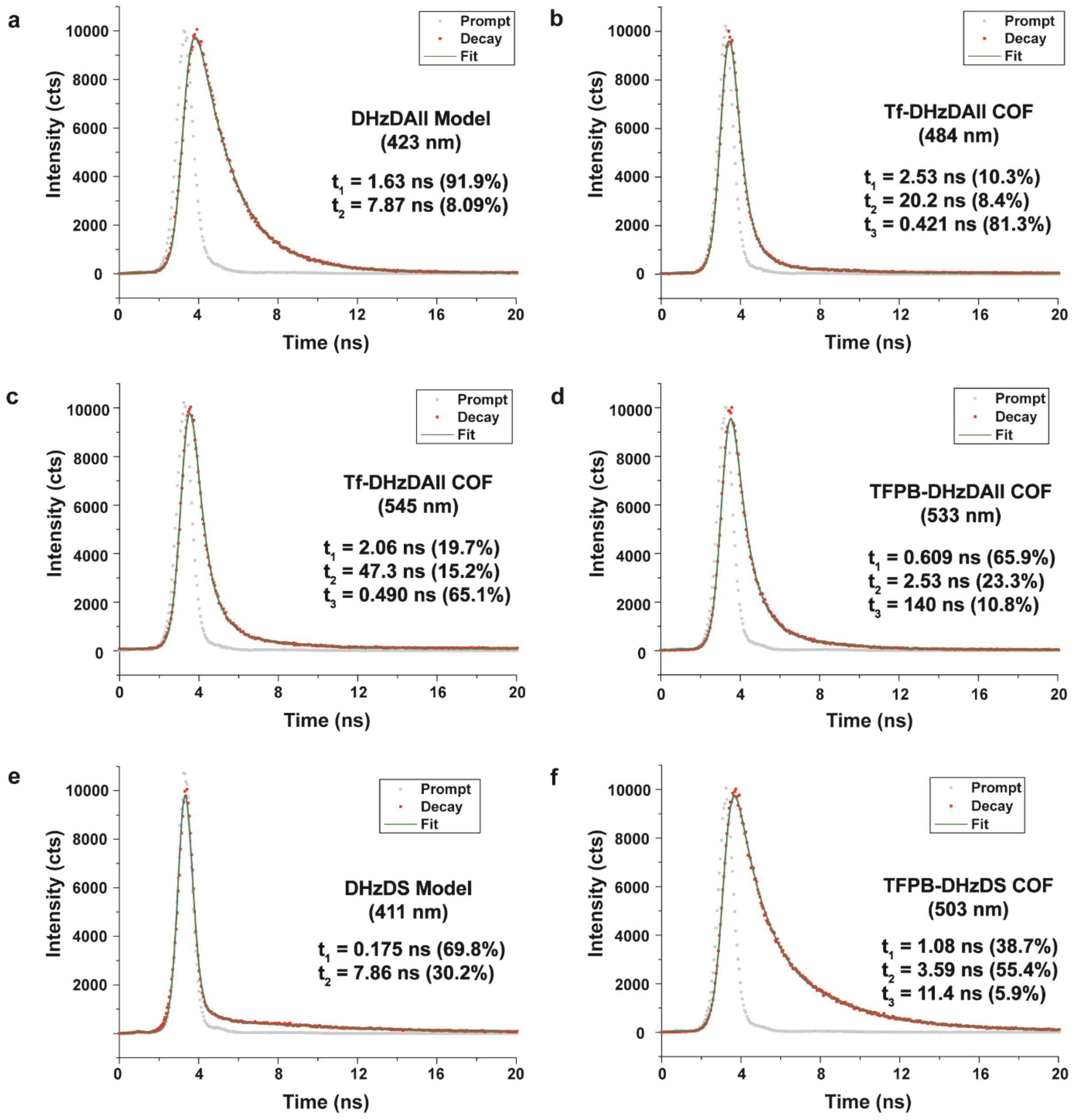

Supplementary Figure 23 | PL lifetime of various COFs and model compounds.

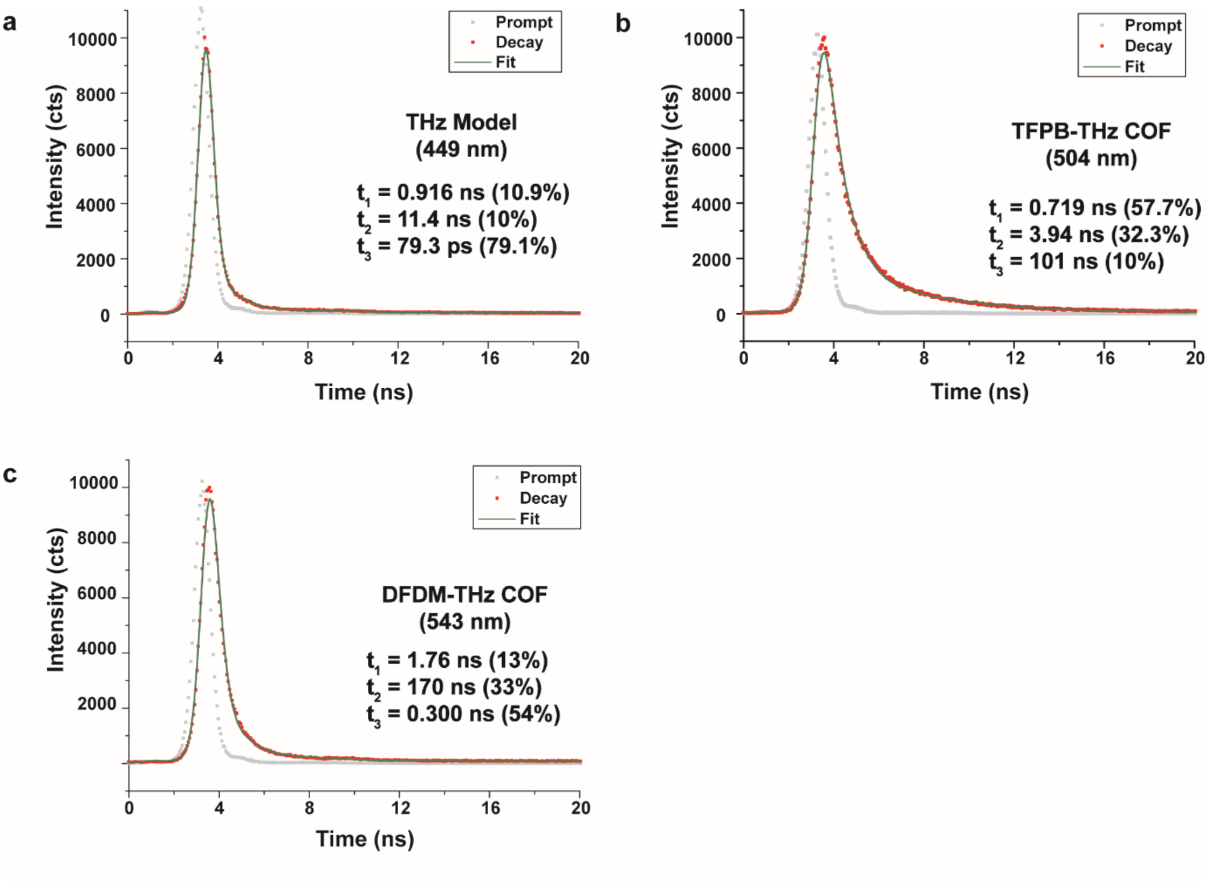

Supplementary Figure 24 | PL lifetime of various COFs and model compounds.

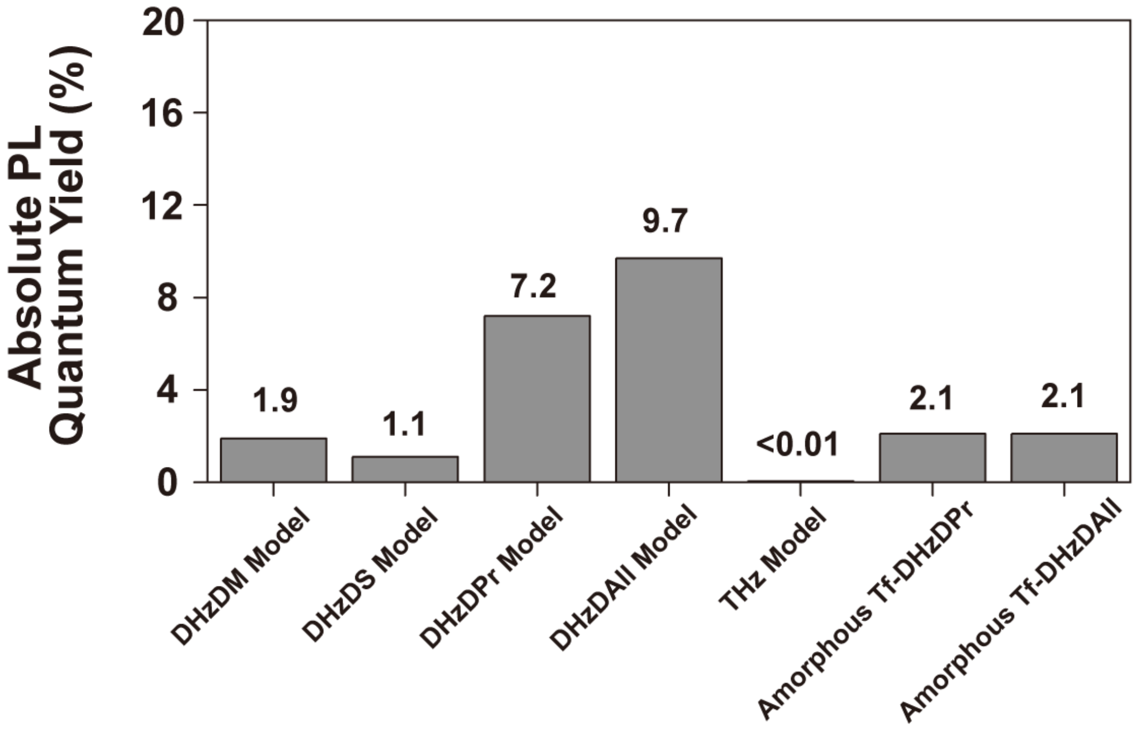

Supplementary Figure 25 | PL Quantum yields of model compounds and amorphous polymer of Tf-DHzDPr and Tf-DHzDAll.

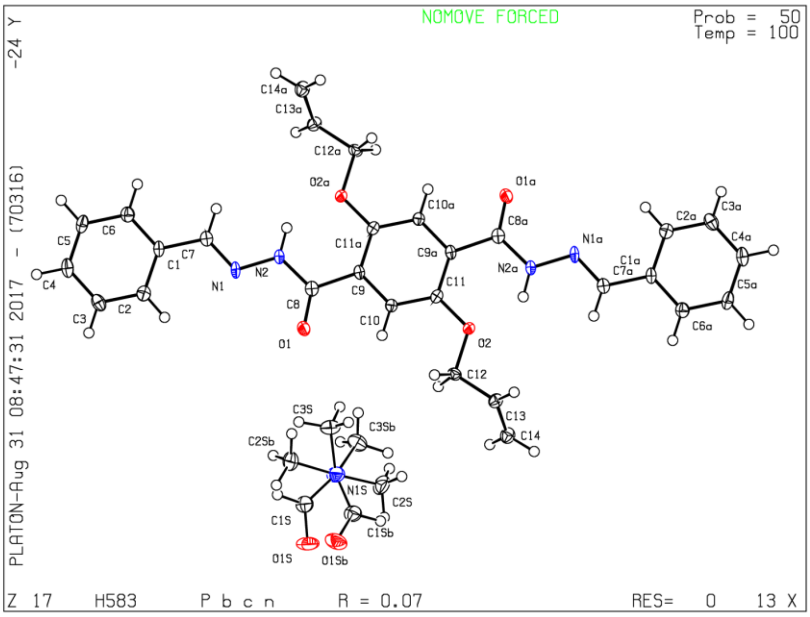

Supplementary Figure 26 | Single crystal structure of DHzDAll Model compound.

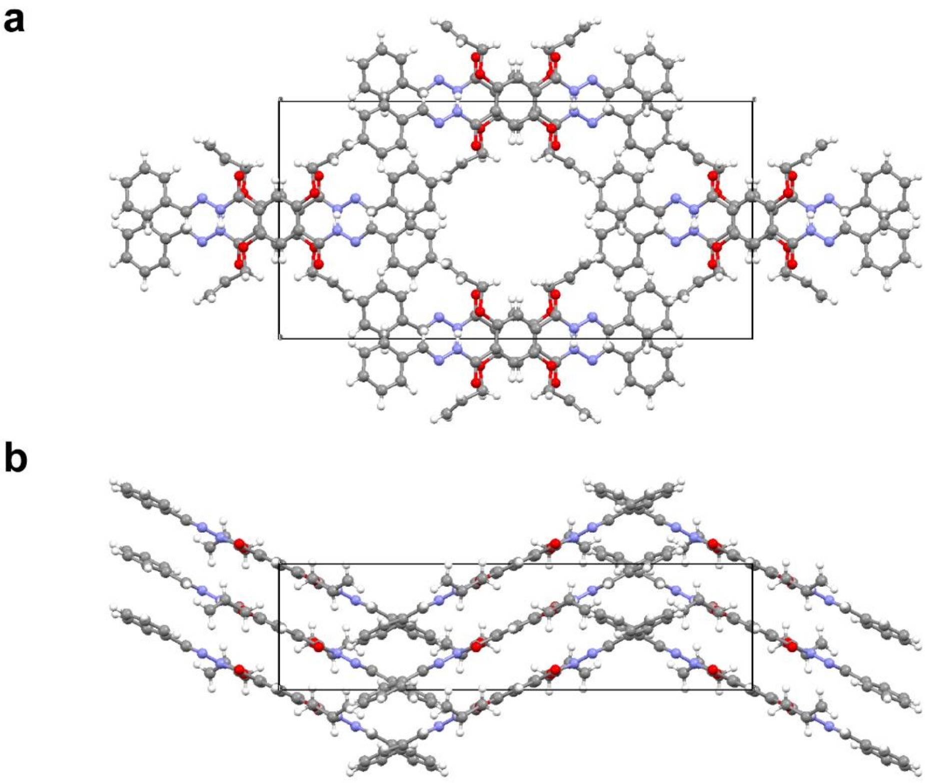

Supplementary Figure 27 | Crystal packing of DHzDAll Model compound.

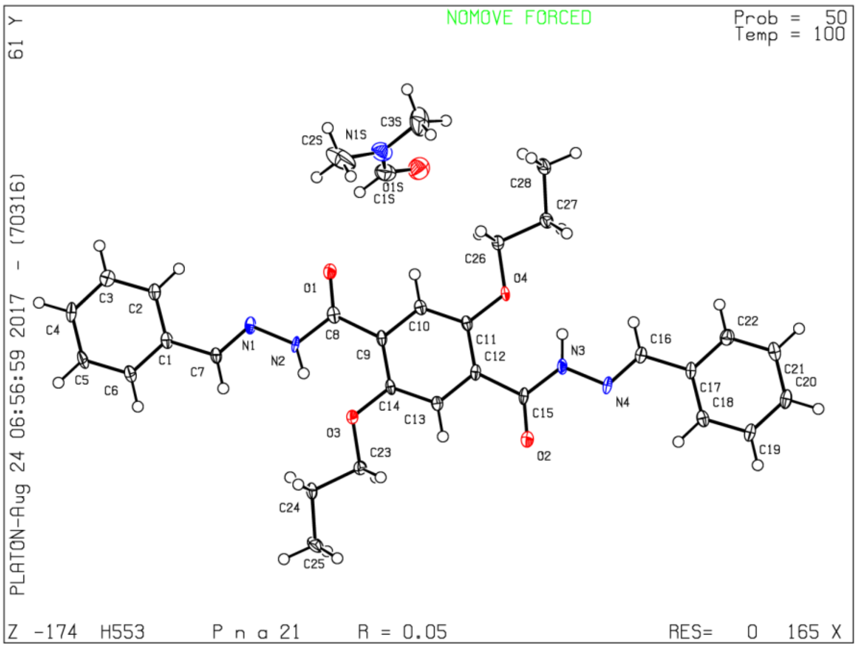

Supplementary Figure 28 | Single crystal structure of DHzDPr Model compound.

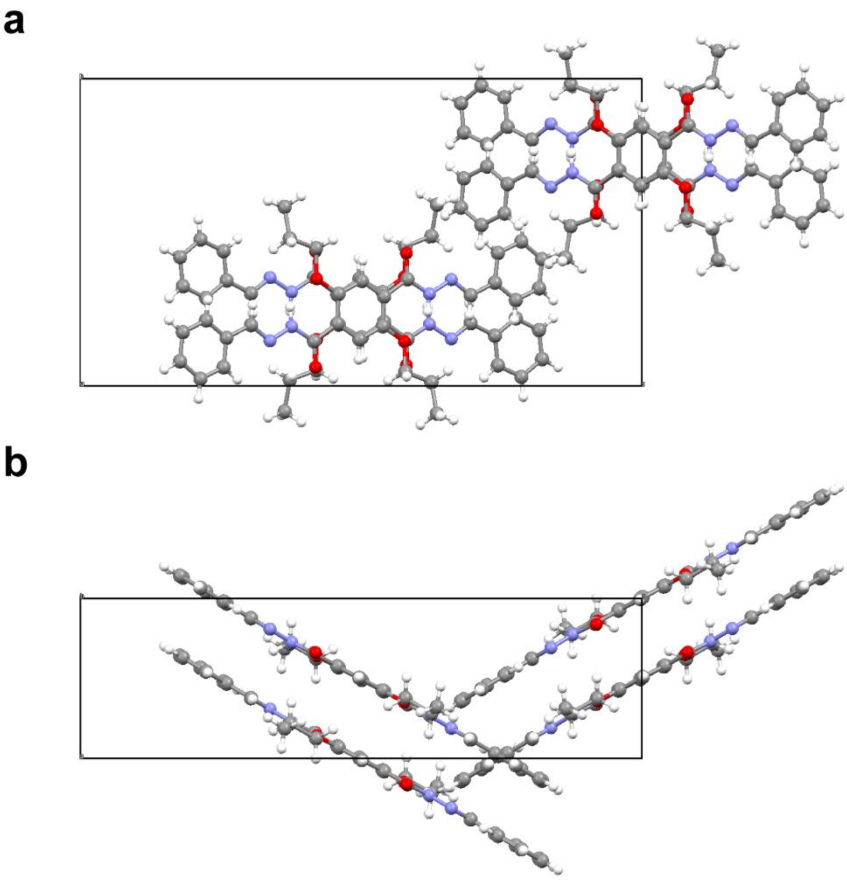

Supplementary Figure 29 | Crystal packing of DHzDPr Model compound.

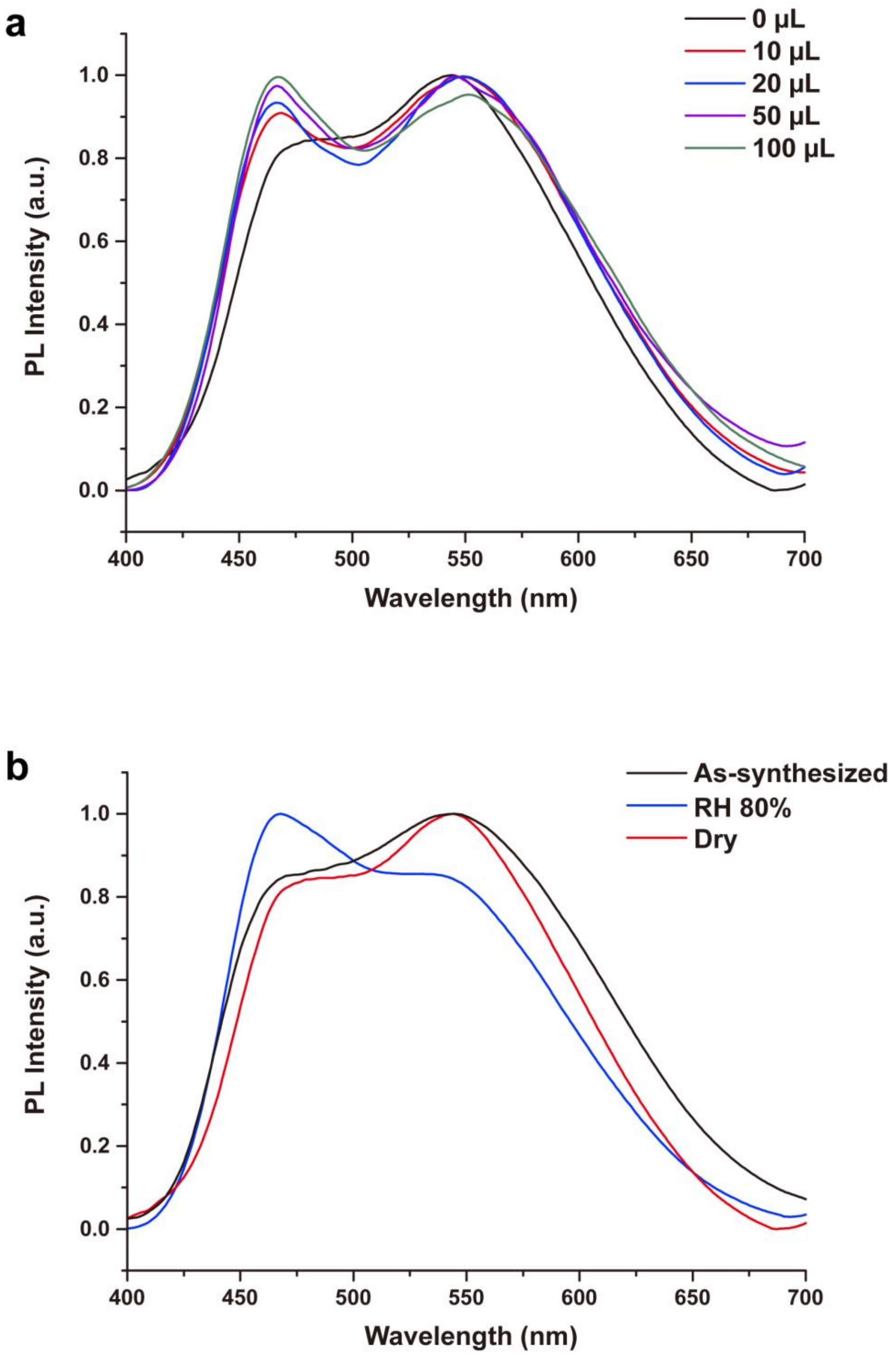

Supplementary Figure 30 | Water effect on the dual-emissive PL of Tf-DHzDAll COF. **a**, PL of Tf-DHzDAll (5 mg) wetted with various amount of water. **b**, PL of the as-synthesized Tf-DHzDAll COF, exposed to relative humidity (RH) of 80% for a week, and dried solid.

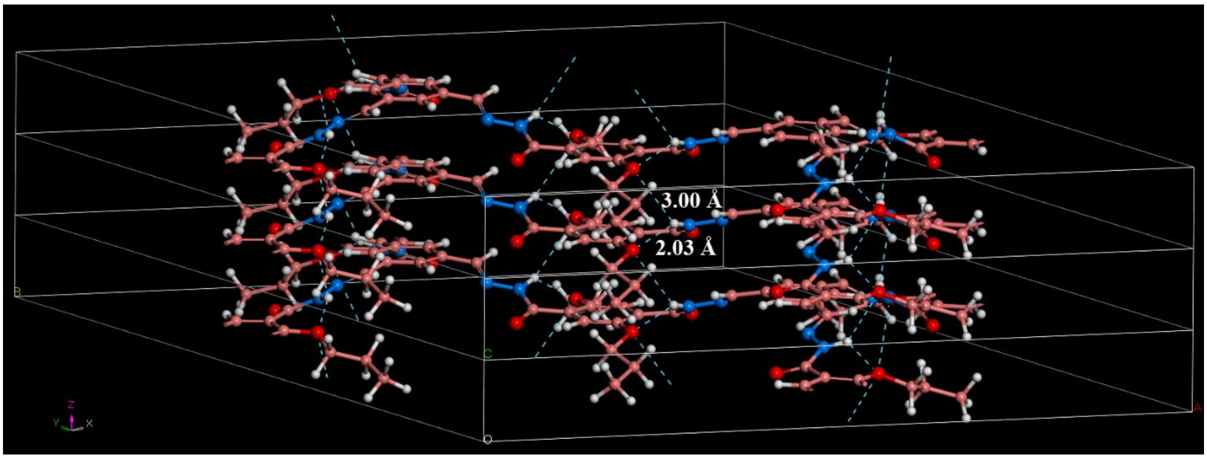

Supplementary Figure 31 | Lattice structure of Tf-DHzDPr COF with intralayer hydrogen bonding of 2.03 Å and interlayer hydrogen bonding of 3.00 Å. (C, pink; N; blue; O, red; H, white; hydrogen bonding, light blue dash line.)

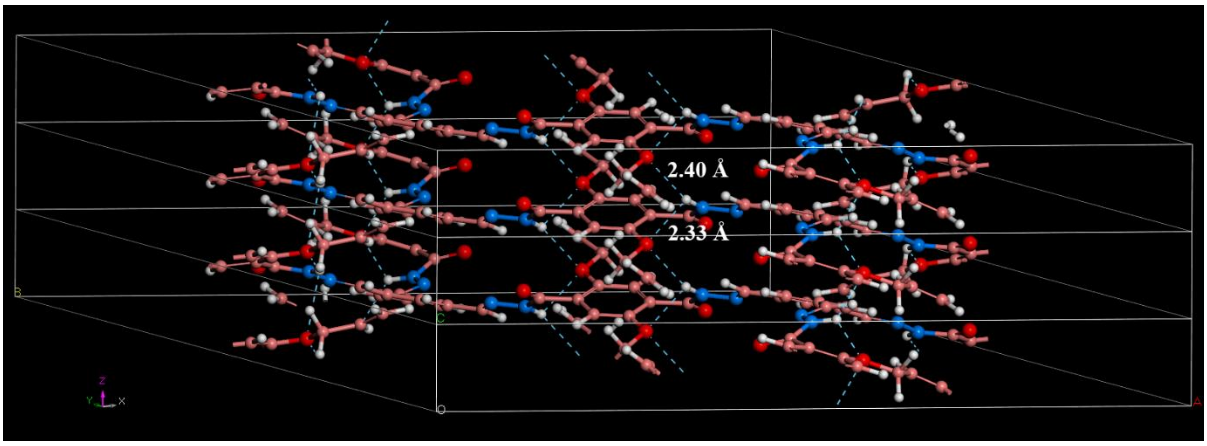

Supplementary Figure 32 | Lattice structure of Tf-DHzDAll COF with intralayer hydrogen bonding of 2.33 Å and interlayer hydrogen bonding of 2.40 Å. (C, pink; N; blue; O, red; H, white; hydrogen bonding, light blue dash line.)

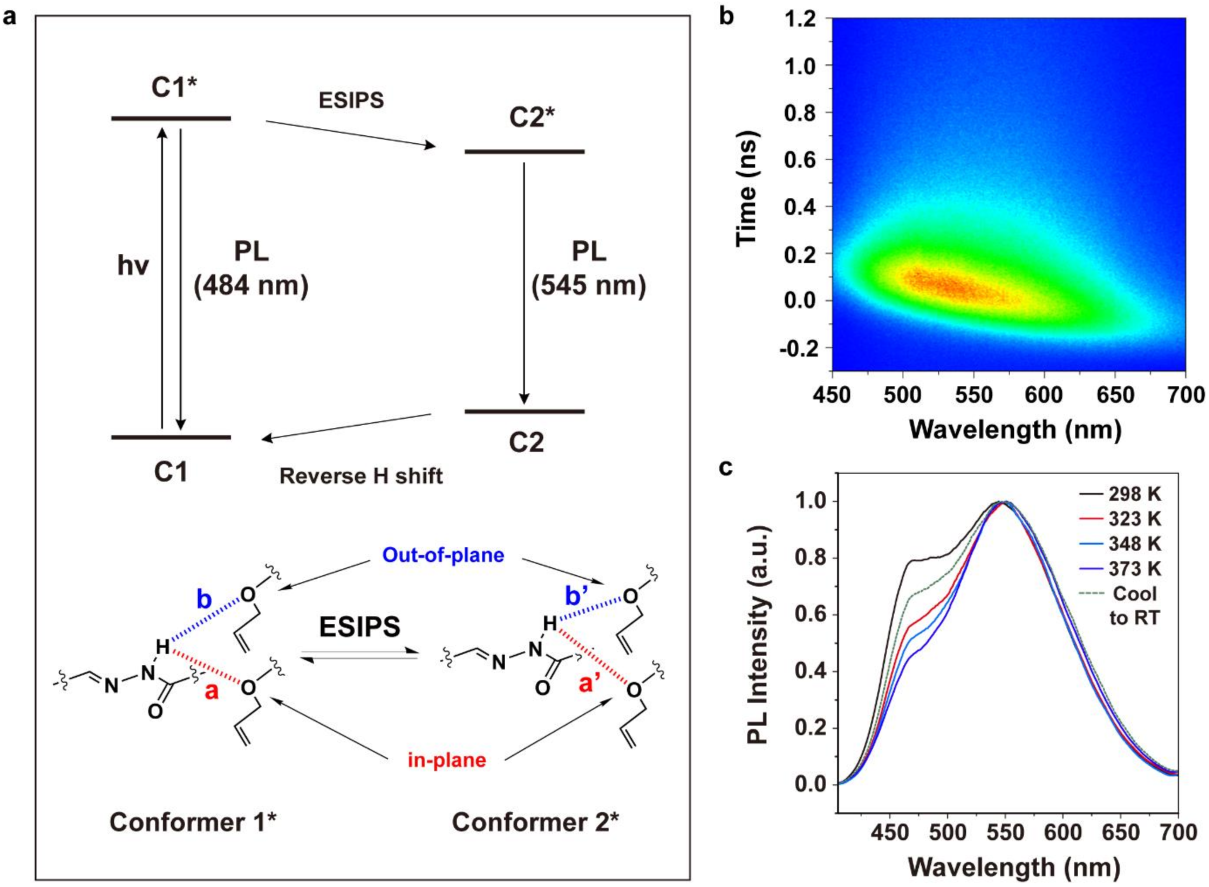

Supplementary Figure 33 | Study of the mechanism for dual emission. a, Schematic Energy diagram of the proposed COF-triggered ESIPS process. **b**, 2D plot of the time-resolved PL spectra of the Tf-DHzDAll COF (excitation wavelength 380 nm; PL intensity: 0 to maximum, dark blue to red). **c**, Temperature-dependent PL study of the Tf-DHzDAll COF.

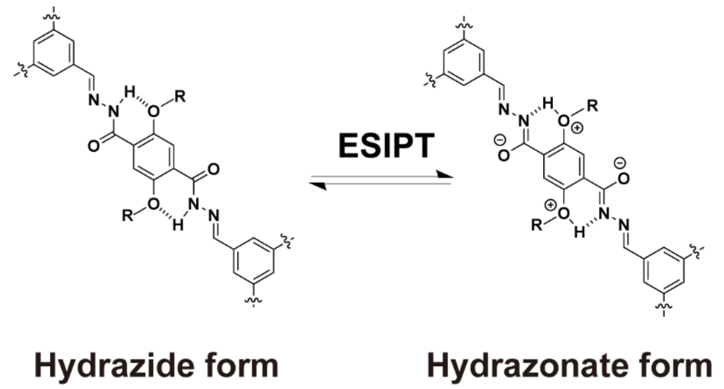

Supplementary Figure 34 | Proposed ESIPT pathway based on hydrazide and hydrazonate tautomerization.

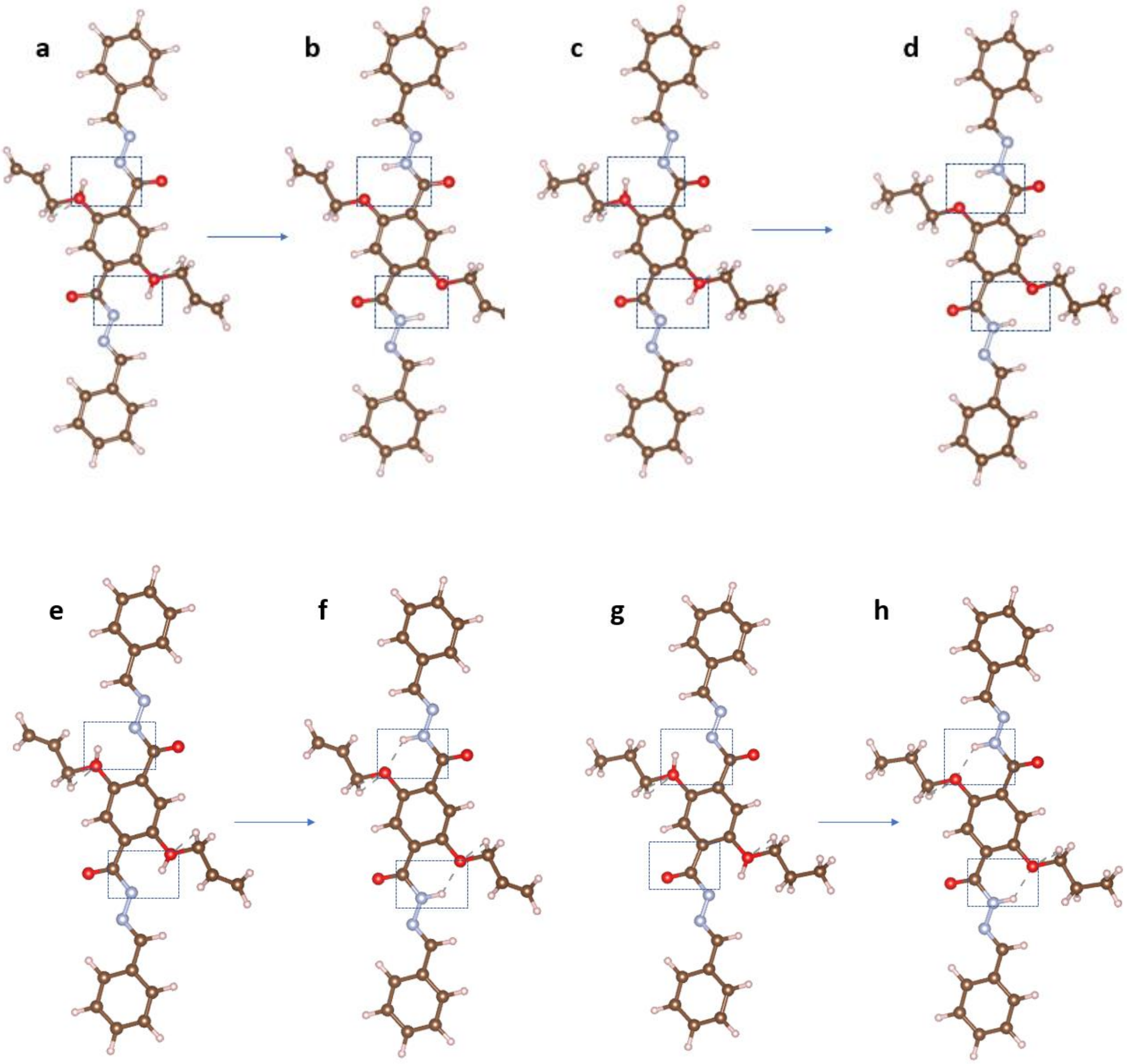

Supplementary Figure 35 | Ground-state and excited-state structures. **a**, Ground-state structure of DHzDAll molecular fragment after artificial proton transfer. **b**, Optimized ground-state structure of DHzDAll molecular fragment. **c**, Ground-state structure of DHzDPr molecular fragment after artificial proton transfer. **d**, Optimized ground-state structure of DHzDPr molecular fragment. **e**, Excited-state structure of DHzDAll molecular fragment after artificial proton transfer. **f**, Optimized excited-state structure of DHzDAll molecular fragment. **g**, Excited-state structure of DHzDPr molecular fragment after artificial proton transfer. **h**, Optimized excited-state structure of DHzDPr molecular fragment. (The movement of H from N to O, and from O to N is shown in the dashed box. C, grey; H, white; O, red; N, blue.)

Supplementary Tables

Supplementary Table 1 | Band gaps, absorption maxima, emission maxima and absolute PLQY of various COFs and model compounds.

	**Band Gap (eV)**	**Absorption λ_{max} (nm)**	**Emission λ_{max} (nm)**	**Absolute PLQY (%)**
**DHzDM Model**	2.95 (421 nm)	378	411	1.9
**DHzDPr Model**	2.89 (429 nm)	368	425[a]	7.2
**DHzDS Model**	2.96 (420 nm)	373	411	1.1
**DHzDAll Model**	2.87 (433 nm)	371	423[a]	9.7
**THz Model**	2.82 (440 nm)	291, 338	449	<0.01
**Tf-DHzDM COF**	2.76 (450 nm)	390	456	8.2
**Tf-DHzDPr COF**	2.72 (456 nm)	388	456	11.9
**Tf-DHzDAll COF**	2.63 (472 nm)	387	484, 545	3.9
**TFPB-DHzDM COF**	2.61 (475 nm)	369	495	6.6
**TFPB-DHzDPr COF**	2.63 (472 nm)	378	474	14.4
**TFPB-DHzDS COF**	2.59 (480 nm)	386	503	16.3
**TFPB-DHzDAll COF**	2.56 (485 nm)	404	490, 533	3.6
**TFPB-THz COF**	2.41 (516 nm)	292, 380	504	2.4
**DFDM-THz COF**	2.18 (570 nm)	300, 377	543	0.4

[a] The data was recorded as single crystal form.

Supplementary Table 2 | Crystal data of DHzDAll Model.

**CCDC deposition number**	1818845	
**Chemical formula**	$C_{31}H_{33}N_5O_5$	
**Formula weight**	555.62 g mol^{-1}	
**Temperature**	100(2) K	
**Wavelength**	1.54178 Å	
**Crystal size**	0.048 x 0.058 x 0.472 mm	
**Crystal system**	orthorhombic	
**Space group**	P b c n	
**Unit cell dimensions**	a = 27.5137(8) Å	α = 90°
	b = 13.8315(5) Å	β = 90°
	c = 7.3394(2) Å	γ = 90°
**Volume**	2793.05(15) $Å^3$	
**Z**	4	
**Density (calculated)**	1.321 g cm^{-3}	
**Absorption coefficient**	0.744 mm^{-1}	
**F(000)**	1176	

**Theta range for data collection**	5.79 to 66.57°	
**Index ranges**	-32<=h<=28, -16<=k<=14, -8<=l<=7	
**Reflections collected**	16777	
**Independent reflections**	2466 [R(int) = 0.0837]	
**Coverage of independent reflections**	99.6%	
**Absorption correction**	Multi-Scan	
**Max. and min. transmission**	0.9650 and 0.7200	
**Structure solution technique**	direct methods	
**Structure solution program**	SHELXS-97 (Sheldrick 2008)	
**Refinement method**	Full-matrix least-squares on F^2	
**Refinement program**	SHELXL-2014/7 (Sheldrick, 2014)	
**Function minimized**	$\Sigma w(F_o^2 - F_c^2)^2$	
**Data / restraints / parameters**	2466 / 0 / 211	
**Goodness-of-fit on F^2**	1.209	
**Final R indices**	2093 data; I>2σ(I)	R1 = 0.0699, wR2 = 0.2123
	all data	R1 = 0.0800, wR2 = 0.2195
**Weighting scheme**	$w=1/[\sigma^2(F_o^2)+(0.0941P)^2+4.9091P]$ where $P=(F_o^2+2F_c^2)/3$	
**Extinction coefficient**	0.0019(4)	
**Largest diff. peak and hole**	0.345 and -0.273 $eÅ^{-3}$	
**R.M.S. deviation from mean**	0.073 $eÅ^{-3}$	

Supplementary Table 3 | Crystal data of DHzDPr Model.

**CCDC deposition number**	1818846	
**Chemical formula**	$C_{31}H_{37}N_5O_5$	
**Formula weight**	559.65 g mol^{-1}	
**Temperature**	100(2) K	
**Wavelength**	1.54178 Å	
**Crystal size**	0.082 x 0.101 x 0.452 mm	
**Crystal system**	orthorhombic	
**Space group**	P n a 21	
**Unit cell dimensions**	a = 7.5394(2) Å	α = 90°
	b = 14.4953(5) Å	β = 90°
	c = 26.6114(8) Å	γ = 90°
**Volume**	2908.25(15) Å^3	
**Z**	4	
**Density (calculated)**	1.278 g cm^{-3}	
**Absorption coefficient**	0.715 mm^{-1}	
**F(000)**	1192	

**Theta range for data collection**	3.32 to 74.71°	
**Index ranges**	-9<=h<=8, -18<=k<=17, -32<=l<=33	
**Reflections collected**	21912	
**Independent reflections**	5653 [R(int) = 0.0666]	
**Coverage of independent reflections**	99.7%	
**Absorption correction**	Multi-Scan	
**Max. and min. transmission**	0.9440 and 0.7380	
**Structure solution technique**	direct methods	
**Structure solution program**	SHELXS-97 (Sheldrick 2008)	
**Refinement method**	Full-matrix least-squares on F^2	
**Refinement program**	SHELXL-2014/7 (Sheldrick, 2014)	
**Function minimized**	Σ w(F_o^2 - F_c^2)^2	
**Data / restraints / parameters**	5653 / 1 / 381	
**Goodness-of-fit on F^2**	1.028	
**Final R indices**	4768 data; I>2σ(I)	R1 = 0.0473, wR2 = 0.1142
	all data	R1 = 0.0598, wR2 = 0.1224
**Weighting scheme**	w=1/[$\sigma^2(F_o^2)+(0.0688P)^2+0.2498P$] where P=($F_o^2+2F_c^2$)/3	
**Absolute structure parameter**	0.3(3)	
**Largest diff. peak and hole**	0.195 and -0.321 eÅ^{-3}	
**R.M.S. deviation from mean**	0.58 eÅ^{-3}	

Supplementary Table 4 | Atomistic coordinates for vdW-DF2 optimized unit cell parameters for Tf-DHzDPr COF (space group *P1*, a = b = 29.3729 Å, c = 3.4420 Å, α = β = 90°, and γ = 120°).

Atom	x	y	z
C1	0.94608	0.46223	0.50123
C2	0.53777	0.48384	0.5013
C3	0.51616	0.05394	0.5012
C4	0.98427	0.44737	0.48942
C5	0.55263	0.53688	0.48969
C6	0.46311	0.01575	0.48959
C7	0.03744	0.4838	0.48633
C8	0.51621	0.55363	0.48649
C9	0.44637	0.96258	0.48642
C10	0.89092	0.41716	0.53225
C11	0.58284	0.47375	0.53226
C12	0.52623	0.1091	0.53217
N13	0.8554	0.42954	0.68922
N14	0.57047	0.42584	0.68913
N15	0.57416	0.14462	0.68923
N16	0.80526	0.39133	0.76087
N17	0.60869	0.41393	0.76076
N18	0.58607	0.19475	0.76084
C19	0.77549	0.40722	0.91967
C20	0.59279	0.36826	0.91962
C21	0.63175	0.22452	0.9197
C22	0.72048	0.36966	-0.01766
C23	0.63036	0.35083	-0.01768
C24	0.64919	0.27953	-0.01764
O25	0.87874	0.3712	0.43752
O26	0.62881	0.50753	0.43751
O27	0.49248	0.12128	0.43751
C28	0.70269	0.31563	-0.01061
C29	0.68439	0.38707	-0.01069
C30	0.61295	0.29732	-0.0106
O31	0.92432	0.53099	0.47362
O32	0.46901	0.39331	0.47352
O33	0.60669	0.07569	0.47361
C34	0.94109	0.58373	0.33484
C35	0.41628	0.35736	0.33451
C36	0.64264	0.05891	0.3346
H37	0.96979	0.40542	0.48712
H38	0.59459	0.56436	0.48765
H39	0.43564	0.03023	0.48751
H40	0.86778	0.4681	0.76868
H41	0.53192	0.39968	0.76866
H42	0.60033	0.13225	0.76871
H43	0.78987	0.44896	-0.01599
H44	0.55106	0.3409	-0.01596

H45	0.65911	0.21014	-0.01592
H46	0.69759	0.42889	-0.01901
H47	0.57113	0.26871	-0.01891
H48	0.73131	0.30243	-0.01888
C49	0.05394	0.53775	0.50155
C50	0.46224	0.51617	0.5014
C51	0.48382	0.94607	0.50131
C52	0.01576	0.55261	0.49011
C53	0.44739	0.46313	0.48988
C54	0.53686	0.98426	0.48984
C55	0.9626	0.5162	0.48663
C56	0.48381	0.44638	0.48654
C57	0.55361	0.03742	0.48652
C58	0.10911	0.58282	0.53235
C59	0.41717	0.52626	0.53234
C60	0.47373	0.89091	0.53215
O61	0.12131	0.62878	0.43749
O62	0.3712	0.49249	0.43757
O63	0.5075	0.87871	0.4374
N64	0.14464	0.57044	0.68903
N65	0.42955	0.57417	0.68925
N66	0.42582	0.85537	0.68893
N67	0.19477	0.60867	0.76071
N68	0.39133	0.58607	0.76094
N69	0.41391	0.80525	0.76059
C70	0.22454	0.59278	0.91963
C71	0.40723	0.63175	0.91978
C72	0.36825	0.77547	0.91949
C73	0.27953	0.63035	-0.01763
C74	0.36965	0.64918	-0.01754
C75	0.35083	0.72048	-0.01773
C76	0.29732	0.68438	-0.01074
C77	0.31563	0.61294	-0.01052
C78	0.38706	0.70268	-0.01062
O79	0.07571	0.46899	0.47323
O80	0.531	0.6067	0.47343
O81	0.3933	0.9243	0.4734
C82	0.05893	0.41627	0.33396
C83	0.58374	0.64265	0.3344
C84	0.35736	0.94109	0.33419
H85	0.03024	0.59458	0.4885
H86	0.40543	0.43565	0.488
H87	0.56434	0.96977	0.488
H88	0.13226	0.5319	0.76873
H89	0.46809	0.60034	0.76884
H90	0.39964	0.86774	0.76858
H91	0.21018	0.55104	-0.0159
H92	0.44896	0.65912	-0.01589

H93	0.34087	0.78983	-0.01612
H94	0.30241	0.57112	-0.01888
H95	0.42888	0.73129	-0.01903
H96	0.26871	0.69759	-0.01922
C97	0.58533	0.69347	0.21852
C98	0.30653	0.89187	0.21824
C99	0.10814	0.41466	0.21798
C100	0.64088	0.73703	0.10355
C101	0.26297	0.90387	0.10337
C102	0.09614	0.35911	0.10318
C103	0.41468	0.30654	0.21861
C104	0.69346	0.10814	0.21874
C105	0.89186	0.58532	0.21898
C106	0.35913	0.26297	0.10361
C107	0.73703	0.09616	0.10369
C108	0.90384	0.64086	0.10385
H109	0.96739	0.59233	0.08666
H110	0.4077	0.37507	0.08632
H111	0.62494	0.03263	0.08639
H112	0.96348	0.61299	0.55941
H113	0.387	0.35048	0.55898
H114	0.64952	0.03651	0.55906
H115	0.03264	0.40773	0.08579
H116	0.59231	0.62493	0.08621
H117	0.3751	0.96738	0.086
H118	0.03651	0.38697	0.55824
H119	0.61302	0.64953	0.55883
H120	0.35047	0.9635	0.55853
H121	0.57055	0.70736	0.45522
H122	0.29264	0.86319	0.45486
H123	0.13683	0.42946	0.45455
H124	0.55768	0.68445	0.97888
H125	0.31555	0.87325	0.97851
H126	0.12676	0.44229	0.97823
H127	0.65992	0.72201	0.90985
H128	0.27798	0.93793	0.90965
H129	0.06208	0.34005	0.90951
H130	0.66634	0.75351	0.35652
H131	0.2465	0.91284	0.35639
H132	0.08716	0.33366	0.3562
H133	0.63941	0.76927	0.95564
H134	0.23073	0.87015	0.95548
H135	0.12985	0.36056	0.9553
H136	0.42946	0.29265	0.45531
H137	0.70735	0.1368	0.45549
H138	0.86321	0.57056	0.45578
H139	0.44234	0.31556	0.97898
H140	0.68444	0.12678	0.97914

H141	0.87321	0.55765	0.97944
H142	0.36062	0.23073	0.95569
H143	0.76927	0.12989	0.95578
H144	0.87012	0.63937	0.95591
H145	0.34009	0.27798	0.90989
H146	0.72201	0.0621	0.90996
H147	0.9379	0.6599	0.91015
H148	0.33368	0.24649	0.35658
H149	0.75352	0.08719	0.35665
H150	0.91282	0.66633	0.35675

Supplementary Table 5 | Atomistic coordinates for vdW-DF2 optimized unit cell parameters for Tf-DHzDAll COF (space group *P1*, a = b = 29.3509 Å, c = 3.4357 Å, α = β = 90°, and γ = 120°).

Atom	x	y	z
C1	0.51435	0.97155	0.53886
C2	0.50882	0.91787	0.50376
N3	0.46105	0.88112	0.34813
N4	0.44542	0.82904	0.33707
C5	0.39649	0.79825	0.26445
C6	0.37422	0.7417	0.28202
O7	0.54401	0.90965	0.59111
C8	0.40642	0.7193	0.24365
O9	0.42504	0.93979	0.73355
C10	0.38349	0.94982	0.84957
H11	0.43369	0.89197	0.30361
H12	0.36944	0.81323	0.21916
H13	0.44728	0.74431	0.16872
C14	0.48515	0.03362	0.67102
C15	0.47421	0.98196	0.64683
H16	0.45579	0.04352	0.75226
C17	0.33547	0.89771	0.93487
C18	0.29221	0.89221	0.09939
H19	0.37596	0.97028	0.61628
H20	0.39657	0.97604	0.09899
H21	0.33746	0.86365	0.84023
H22	0.25825	0.85401	0.14604
H23	0.28897	0.92563	0.19216
C24	0.07711	0.56634	0.96121
C25	0.12775	0.61507	0.84563
N26	0.1687	0.60548	0.79233
N27	0.21625	0.64162	0.64917
C28	0.24787	0.62395	0.59326
C29	0.30111	0.65681	0.45698
O30	0.13028	0.65687	0.78275
C31	0.32151	0.71044	0.37813
O32	0.11401	0.51235	0.14966
C33	0.10803	0.46337	0.29455
H34	0.16237	0.56912	0.85695
H35	0.23593	0.58302	0.65815
C36	0.02009	0.47613	0.15837
C37	0.0702	0.51759	0.08921
H38	0.01314	0.43853	0.2589
H39	0.08671	0.4323	0.08146
H40	0.08429	0.45229	0.55645
C41	0.1623	0.47196	0.36698
C42	0.17237	0.43736	0.53509
H43	0.19414	0.50908	0.2649
H44	0.21213	0.44553	0.5775

H45	0.14146	0.39972	0.6378
H46	0.29632	0.72723	0.40895
C47	0.48484	0.53334	0.55147
C48	0.47851	0.48482	0.44496
C49	0.52106	0.47663	0.43236
C50	0.43533	0.53646	0.56356
N51	0.44241	0.58261	0.40537
N52	0.40594	0.59795	0.42824
C53	0.42032	0.64389	0.29193
C54	0.38603	0.66598	0.3136
O55	0.39449	0.50126	0.69524
C56	0.33325	0.63483	0.42315
H57	0.43935	0.45459	0.36919
H58	0.47781	0.60747	0.28409
H59	0.45924	0.66845	0.16465
O60	0.51621	0.42912	0.31881
C61	0.46547	0.38725	0.17988
H62	0.45251	0.4035	0.94928
H63	0.43619	0.37398	0.41024
C64	0.47322	0.34344	1.03878
C65	0.4414	0.29302	0.12062
H66	0.50612	0.35481	0.84531
H67	0.40797	0.28028	0.31042
H68	0.44781	0.26285	0.9991
H69	0.31826	0.59376	0.48744
C70	0.53523	0.07562	0.59554
C71	0.54072	0.12934	0.62858
N72	0.58923	0.16673	0.76928
N73	0.60533	0.21893	0.76992
C74	0.6546	0.24963	0.83062
C75	0.67751	0.30624	0.80523
O76	0.50503	0.1372	0.54973
C77	0.64617	0.32957	0.84616
O78	0.62449	0.10726	0.40304
C79	0.66625	0.09823	0.27495
H80	0.61669	0.15593	0.81032
H81	0.68145	0.23436	0.87384
H82	0.6054	0.30531	-0.0732
C83	0.56442	0.01352	0.46289
C84	0.57533	0.06513	0.48755
H85	0.5939	0.00382	0.38066
C86	0.71331	0.15117	0.18922
C87	0.7573	0.15861	1.02458
H88	0.67496	0.07775	0.50149
H89	0.65291	0.0725	1.0228
H90	0.70968	0.18441	0.28153
H91	0.79005	0.19755	0.9758
H92	0.76247	0.12631	0.93218

C93	0.97542	0.48064	0.11285
C94	0.92544	0.43246	0.24683
N95	0.88295	0.4401	0.2691
N96	0.83613	0.40462	0.42886
C97	0.80415	0.42216	0.47273
C98	0.75122	0.39023	0.61864
O99	0.92479	0.39263	0.34998
C100	0.73011	0.33664	0.70257
O101	0.93819	0.53378	0.9164
C102	0.94329	0.58263	0.77714
H103	0.88807	0.47532	0.18258
H104	0.81561	0.46248	0.39047
C105	0.0324	0.57075	0.91118
C106	0.98224	0.52914	0.97954
H107	0.03923	0.60832	0.81013
H108	0.96457	0.61353	0.99143
H109	0.96676	0.59434	0.51389
C110	0.88864	0.57339	0.71084
C111	0.87762	0.60739	0.54355
H112	0.85725	0.53633	0.81806
H113	0.83761	0.59874	0.50769
H114	0.90787	0.64504	0.43586
H115	0.75461	0.31907	0.6714
C116	0.57127	0.51799	0.53378
C117	0.57763	0.56657	0.64066
C118	0.53503	0.57478	0.65194
C119	0.62035	0.51407	0.52227
N120	0.61257	0.46777	0.68247
N121	0.64852	0.45179	0.65847
C122	0.63384	0.40596	0.79836
C123	0.66731	0.38294	0.77324
O124	0.66141	0.54841	0.38714
C125	0.7199	0.41312	0.65618
H126	0.61667	0.59684	0.71918
H127	0.57733	0.44353	0.80854
H128	0.59511	0.38209	-0.06961
O129	0.53955	0.62221	0.76338
C130	0.59001	0.66461	0.90089
H131	0.60333	0.64887	0.13267
H132	0.61925	0.67807	0.67015
C133	0.58155	0.70812	0.03964
C134	0.61297	0.75862	-0.04138
H135	0.54832	0.6964	0.23051
H136	0.6467	0.77181	0.77123
H137	0.60597	0.78852	0.07814
H138	0.73538	0.4542	0.58931

Supplementary Table 6 | Optimized ground-state and excited-state bond length of gas-phase DHzDAll and DHzDPr models before and after artificial proton transfer.

Ground state	After artificial proton transfer		Optimized ground-state structures after proton transfer		Optimized ground-state structures before proton transfer	
Bond length (Å)	O-H	N-H	O-H	N-H	O-H	N-H
DHzDAll Model	1.11	2.07	1.95	1.01	1.95	1.00
DHzDPr Model	1.11	2.10	1.89	1.02	1.93	1.00
Excited state	After artificial proton transfer		Optimized excited-state structures after proton transfer		Optimized excited-state structures before proton transfer	
Bond length (Å)	O-H	N-H	O-H	N-H	O-H	N-H
DHzDAll Model	1.11	2.15	1.86	1.02	1.86	1.02
DHzDPr Model	1.11	2.07	1.86	1.02	1.86	1.02

Supplementary Table 7 | Computed absorption and emission energies for single molecule DHzDAll and DHzDPr models.

	CAM-B3LYP		B3LYP	
Model Molecule	DHzDAll Model	DHzDPr Model	DHzDAll Model	DHzDPr Model
Absorption Energy (nm)	299	306	343	346
Emission Energy (nm)	343	343	459	454

Supplementary Table 8 | Fractional atomistic coordinates for Pawley-refined unit cell parameters of Tf-DHzDM COF (space group *P*3, a = b = 29.8993 Å, c = 3.3970 Å, α = β = 90°, and γ = 120°).

Atom	x	y	z
C1	-0.05361	-0.53779	0.58564
C2	-0.01859	-0.55505	0.60325
C3	0.03394	-0.52111	0.63797
C4	-0.10849	-0.57614	0.54649
N5	-0.14222	-0.5646	0.37444
N6	-0.19382	-0.60145	0.35326
C7	-0.22774	-0.59015	0.24224
C8	-0.28162	-0.62952	0.22885
O9	-0.12368	-0.61932	0.66582
C10	-0.29641	-0.68127	0.22693
O11	-0.0706	-0.46786	0.66355
C12	-0.06012	-0.42176	0.46821
H13	-0.0325	-0.59541	0.58327
H14	-0.12992	-0.52949	0.23943
H15	-0.21676	-0.55111	0.17442
H16	-0.30722	-0.57516	0.22888
C17	0.05193	-0.46835	0.62441
C18	0.01679	-0.45121	0.63252
C19	-0.03577	-0.48516	0.61296
C20	0.10698	-0.42976	0.61614
O21	0.12106	-0.38755	0.75277
N22	0.14205	-0.43971	0.44821
N23	0.19345	-0.40208	0.44342
C24	0.22851	-0.41196	0.33663
C25	0.282	-0.37152	0.33164
C26	0.29537	-0.32014	0.33025
O27	0.06899	-0.53842	0.67753
C28	0.05273	-0.58685	0.85645
H29	0.03056	-0.41094	0.66127
H30	0.13093	-0.47372	0.29832
H31	0.21867	-0.4505	0.26153
H32	0.30972	-0.42419	0.32993
H33	-0.0267	-0.40662	0.26864
H34	-0.05346	-0.39189	0.68654
H35	-0.09405	-0.42929	0.29285
H36	0.08468	-0.58404	1.03474
H37	0.04353	-0.61655	0.63042
H38	0.01919	-0.59921	1.05018

Supplementary Table 9 | Fractional atomistic coordinates for Pawley-refined unit cell parameters of Tf-DHzDPr COF (space group *P*3, a = b = 29.3729 Å, c = 3.4420 Å, α = β = 90°, and γ = 120°).

Atom	x	y	z
C1	-0.05279	-0.53542	0.56104
C2	-0.01711	-0.55211	0.54619
C3	0.03565	-0.51729	0.52977
C4	-0.10776	-0.57437	0.5963
N5	-0.14177	-0.56337	0.77272
N6	-0.19349	-0.60057	0.79482
C7	-0.2275	-0.58945	0.91157
C8	-0.28149	-0.62915	0.92688
O9	-0.1227	-0.61749	0.47226
C10	-0.29603	-0.68106	0.92902
O11	-0.07187	-0.46667	0.4809
C12	-0.05588	-0.41727	0.31728
H13	-0.0308	-0.59266	0.55305
H14	-0.12945	-0.52839	0.91209
H15	-0.21652	-0.55031	0.98262
H16	-0.30759	-0.57484	0.92666
C17	0.05279	-0.46458	0.56104
C18	0.01711	-0.44789	0.54619
C19	-0.03565	-0.48271	0.52977
C20	0.10776	-0.42563	0.5963
O21	0.1227	-0.38251	0.47226
N22	0.14177	-0.43663	0.77272
N23	0.19349	-0.39943	0.79482
C24	0.2275	-0.41055	0.91157
C25	0.28149	-0.37085	0.92688
C26	0.29603	-0.31894	0.92902
O27	0.07187	-0.53333	0.4809
C28	0.05588	-0.58273	0.31728
H29	0.03079	-0.40734	0.55305
H30	0.12945	-0.47161	0.91209
H31	0.21652	-0.44969	0.98262
H32	0.30759	-0.42516	0.92666
C33	0.58556	0.68856	0.18808
C34	0.63907	0.72938	0.05535
C35	0.41444	0.31144	0.18808
C36	0.36093	0.27062	0.05535
H37	-0.02999	-0.41084	0.07149
H38	-0.03237	-0.38567	0.51796
H39	0.02999	-0.58916	0.07149
H40	0.03237	-0.61433	0.51796
H41	0.57334	0.70648	0.4111
H42	0.55589	0.6784	-0.03559
H43	0.65836	0.71287	-0.11651
H44	0.66473	0.74911	0.30537
H45	0.6363	0.75937	-0.11305
H46	0.42666	0.29352	0.4111
H47	0.44411	0.3216	-0.03559

H48	0.3637	0.24063	-0.11305
H49	0.34164	0.28713	-0.11651
H50	0.33527	0.25089	0.30537

Supplementary Table 10 | Fractional atomistic coordinates for Pawley-refined unit cell parameters of Tf-DHzDAll COF (space group *P3*, a = b = 29.3509 Å, c = 3.4357 Å, α = β = 90°, and γ = 120°).

Atom	x	y	z
C1	-0.05226	-0.53503	0.48633
C2	-0.01711	-0.55062	0.40874
C3	0.03519	-0.51624	0.40819
C4	-0.10672	-0.57378	0.48295
N5	-0.14246	-0.56303	0.34955
N6	-0.19359	-0.60024	0.35281
C7	-0.22864	-0.58964	0.27332
C8	-0.28209	-0.62933	0.27459
O9	-0.11963	-0.61648	0.59452
C10	-0.29625	-0.68058	0.27388
O11	-0.07106	-0.46943	0.67084
C12	-0.05481	-0.42038	0.83122
H13	-0.03119	-0.59006	0.3466
H14	-0.13194	-0.52788	0.2271
H15	-0.21878	-0.55099	0.21324
H16	-0.30844	-0.5761	0.27229
C17	0.0523	-0.46512	0.49699
C18	0.01715	-0.44953	0.57431
C19	-0.03513	-0.48394	0.57609
C20	0.10674	-0.42634	0.50009
O21	0.11964	-0.38373	0.3863
N22	0.14247	-0.43696	0.63584
N23	0.1936	-0.39976	0.63213
C24	0.22864	-0.41033	0.71307
C25	0.28208	-0.37066	0.71157
C26	0.29625	-0.3194	0.71228
O27	0.07113	-0.5309	0.31728
C28	0.0549	-0.57981	0.15387
H29	0.03123	-0.41008	0.63603
H30	0.13192	-0.47198	0.76102
H31	0.21876	-0.44896	0.7745
H32	0.30842	-0.42391	0.71395
C33	0.58204	0.68228	0.01111
C34	0.62225	0.72773	0.0736
C35	0.41795	0.31799	0.97489
C36	0.37392	0.2757	1.02997
H37	-0.03412	-0.38951	0.61795
H38	-0.02759	-0.41274	1.0677
H39	0.02837	-0.58669	-0.08601

H40	0.03325	-0.61104	0.36271
H41	0.55012	0.67978	-0.14893
H42	0.65525	0.732	0.2285
H43	0.62241	0.76122	-0.03361
H44	0.45257	0.31818	1.04798
H45	0.3732	0.24203	1.13958
H46	0.33849	0.27436	0.96771

Supplementary Table 11 | Fractional atomistic coordinates for Pawley-refined unit cell parameters of TFPB-DHzDM COF (space group *P3*, a = b = 44.8692 Å, c = 3.3266 Å, α = β = 90°, and γ = 120°).

Atom	x	y	z
C1	-0.03573	0.48483	0.53195
C2	-0.02036	0.46516	0.62073
C3	0.01537	0.4807	0.59713
C4	-0.0732	0.47035	0.54809
O5	-0.08451	0.48825	0.66527
N6	-0.09523	0.43788	0.42293
N7	-0.13046	0.42522	0.4373
C8	-0.15222	0.39434	0.33122
C9	-0.18903	0.3821	0.34052
C10	-0.20152	0.40342	0.46105
C11	-0.23647	0.3914	0.46922
C12	-0.26009	0.35797	0.34305
C13	-0.247	0.33696	0.21753
C14	-0.21206	0.34872	0.22307
C15	-0.29778	0.34529	0.3421
C16	-0.3102	0.3682	0.34174
O17	-0.04147	0.43095	0.74128
H18	-0.0861	0.42294	0.2963
H19	-0.14336	0.37738	0.23028
H20	-0.18433	0.42927	0.55806
H21	-0.24444	0.40845	0.58719
H22	-0.26319	0.31167	0.10085
H23	-0.20293	0.33195	0.12435
H24	-0.29242	0.39502	0.34108
C25	0.03586	0.5153	0.50015
C26	0.02044	0.5349	0.40824
C27	-0.01529	0.51938	0.43346
C28	0.07339	0.5298	0.48955
O29	0.08476	0.5116	0.3864
N30	0.09543	0.56264	0.60285
N31	0.13062	0.575	0.60027
C32	0.1525	0.60716	0.63438
C33	0.18919	0.61909	0.63718
C34	0.20131	0.59721	0.74429
C35	0.2362	0.60896	0.75352

C36	0.26012	0.64262	0.63729
C37	0.24729	0.66415	0.52351
C38	0.21243	0.65274	0.53335
C39	0.29781	0.655	0.63541
C40	0.30997	0.63187	0.63488
O41	0.04144	0.56895	0.28146
H42	0.08643	0.57823	0.71255
H43	0.14389	0.62554	0.64757
H44	0.18383	0.57122	0.83426
H45	0.24381	0.59142	0.86405
H46	0.26358	0.68967	0.41443
H47	0.2035	0.66994	0.44564
H48	0.29203	0.60514	0.63401
C49	1.0266	1.58825	0.13524
C50	0.97328	1.41169	0.8912
H51	0.02781	0.46612	0.65619
H52	-0.02779	0.53391	0.37406
H53	1.01755	1.59836	0.3753
H54	1.00485	1.57284	-0.06369
H55	1.04652	1.61062	-0.02896
H56	0.98244	1.40152	0.65279
H57	0.95329	1.38937	1.0554
H58	0.99493	1.42713	1.09146

Supplementary Table 12 | Fractional atomistic coordinates for Pawley-refined unit cell parameters of TFPB-DHzDPr COF (space group *P3*, a = b = 42.2841 Å, c = 3.5589 Å, α = β = 90°, and γ = 120°).

Atom	x	y	z
C1	-0.03613	0.48472	0.22651
C2	-0.02095	0.46381	0.21679
C3	0.01506	0.47945	0.21378
C4	-0.07391	0.47008	0.24787
O5	-0.08666	0.48598	0.10659
N6	-0.09461	0.43972	0.43207
N7	-0.13017	0.42655	0.44548
C8	-0.1508	0.39707	0.59988
C9	-0.18794	0.38439	0.61504
C10	-0.20152	0.40468	0.48521
C11	-0.2368	0.39213	0.49459
C12	-0.25952	0.35927	0.64523
C13	-0.24543	0.33934	0.78129
C14	-0.21016	0.35159	0.76087
C15	-0.29746	0.34594	0.65671
C16	-0.31045	0.36863	0.65842
O17	-0.04206	0.42793	0.18045
H18	-0.08408	0.42728	0.58576
H19	-0.14075	0.38157	0.71903

H20	-0.18497	0.43003	0.36729
H21	-0.24594	0.40809	0.37013
H22	-0.2612	0.3146	0.91462
H23	-0.20013	0.33568	0.86696
H24	-0.29282	0.39585	0.65846
C25	0.03613	0.51528	0.22651
C26	0.02095	0.53619	0.21679
C27	-0.01506	0.52056	0.21378
C28	0.07391	0.52992	0.24786
O29	0.08666	0.51402	0.10659
N30	0.09461	0.56028	0.43207
N31	0.13018	0.57345	0.44548
C32	0.1508	0.60293	0.59988
C33	0.18794	0.61562	0.61504
C34	0.20152	0.59532	0.4852
C35	0.2368	0.60787	0.49459
C36	0.25952	0.64073	0.64522
C37	0.24543	0.66066	0.78129
C38	0.21016	0.64841	0.76087
C39	0.29746	0.65406	0.6567
C40	0.31045	0.63137	0.65842
O41	0.04206	0.57207	0.18045
H42	0.08408	0.57272	0.58576
H43	0.14075	0.61843	0.71903
H44	0.18497	0.56997	0.36729
H45	0.24594	0.59191	0.37013
H46	0.2612	0.6854	0.91462
H47	0.20013	0.66432	0.86696
H48	0.29282	0.60415	0.65846
C49	1.03052	1.59342	0.35385
C50	0.96948	1.40658	0.35386
H51	0.02691	0.46362	0.20021
H52	-0.02691	0.53638	0.20021
C53	1.06169	1.62753	0.47591
C54	1.05186	1.65403	0.60479
C55	0.93831	1.37247	0.47591
C56	0.94814	1.34597	0.60479
H57	1.01405	1.58029	0.60224
H58	1.014	1.59803	0.15863
H59	0.98595	1.41971	0.60224
H60	0.986	1.40197	0.15863
H61	1.08124	1.6396	0.25076
H62	1.07535	1.62177	0.69837
H63	1.02813	1.64215	0.77903
H64	1.04684	1.66626	0.35778
H65	1.0735	1.67481	0.76742
H66	0.91876	1.3604	0.25076
H67	0.92465	1.37823	0.69837

H68	0.95316	1.33374	0.35778
H69	0.9265	1.32519	0.76743
H70	0.97187	1.35785	0.77903

Supplementary Table 13 | Fractional atomistic coordinates for Pawley-refined unit cell parameters of TFPB-DHzDAll COF (space group *P3*, a = b = 41.8503 Å, c = 3.4997 Å, α = β = 90°, and γ = 120°).

Atom	x	y	z
C1	-0.0365	0.48434	0.34126
C2	-0.02174	0.4631	0.34781
C3	0.01415	0.47828	0.33558
C4	-0.07411	0.47042	0.37591
O5	-0.08677	0.48624	0.23359
N6	-0.09476	0.44077	0.56931
N7	-0.13025	0.42777	0.5827
C8	-0.15083	0.39865	0.74154
C9	-0.18796	0.38577	0.75372
C10	-0.20177	0.4056	0.61874
C11	-0.23706	0.39274	0.62518
C12	-0.25954	0.36003	0.77683
C13	-0.24524	0.34059	0.91788
C14	-0.20998	0.35316	0.90144
C15	-0.29747	0.34631	0.785
C16	-0.31082	0.36862	0.78614
O17	-0.04324	0.42722	0.33843
H18	-0.08432	0.4286	0.72663
H19	-0.14077	0.38342	0.86483
H20	-0.18538	0.43079	0.49949
H21	-0.24642	0.40832	0.49827
H22	-0.26088	0.31599	1.05175
H23	-0.1998	0.3376	1.01142
H24	-0.29346	0.39584	0.78592
C25	0.03544	0.51391	0.31189
C26	0.02069	0.5349	0.26991
C27	-0.01518	0.51984	0.29431
C28	0.07313	0.52835	0.31734
O29	0.08523	0.51191	0.17844
N30	0.09441	0.55922	0.48524
N31	0.12992	0.57232	0.48724
C32	0.1509	0.60199	0.63228
C33	0.18804	0.61487	0.63555
C34	0.2016	0.59491	0.49772
C35	0.23687	0.60766	0.49894
C36	0.25961	0.6404	0.64839
C37	0.24557	0.66002	0.7912
C38	0.21032	0.64756	0.77972
C39	0.29751	0.6539	0.65374

C40	0.31065	0.63142	0.65456
O41	0.04249	0.57016	0.19765
H42	0.08432	0.57239	0.63034
H43	0.14113	0.61754	0.7534
H44	0.18501	0.56966	0.38076
H45	0.24602	0.59196	0.37054
H46	0.26142	0.68466	0.92338
H47	0.20035	0.66323	0.89182
H48	0.29316	0.60423	0.65459
C49	1.0282	1.58964	0.0441
C50	0.96879	1.40662	0.51558
H51	0.02578	0.46232	0.34074
H52	-0.0268	0.53577	0.28179
C53	1.0567	1.62341	-0.09442
C54	1.05149	1.64947	-0.17014
C55	0.93848	1.37371	0.64682
C56	0.9346	1.34316	0.558
H57	1.01299	1.59434	0.2567
H58	1.01065	1.57539	-0.18983
H59	0.98521	1.42022	0.76015
H60	0.98484	1.40143	0.31897
H61	1.08175	1.62635	-0.14949
H62	1.02654	1.6471	-0.12676
H63	1.07232	1.67343	-0.27794
H64	0.91908	1.37479	0.81948
H65	0.9123	1.31995	0.65808
H66	0.9535	1.34097	0.3877

Supplementary Table 14 | Fractional atomistic coordinates for Pawley-refined unit cell parameters of TFPB-DHzDS COF (space group *P3*, a = b = 44.3328 Å, c = 3.3890 Å, α = β = 90°, and γ = 120°).

Atom	x	y	z
C1	-0.03596	0.48877	0.63716
C2	-0.02073	0.46803	0.67799
C3	0.01533	0.48377	0.66983
C4	-0.07378	0.4739	0.60869
O5	-0.08674	0.49027	0.73532
N6	-0.09427	0.4427	0.43438
N7	-0.12988	0.42921	0.41614
C8	-0.1503	0.39893	0.27356
C9	-0.18748	0.38588	0.25415
C10	-0.2013	0.40641	0.37123
C11	-0.23664	0.39351	0.36073
C12	-0.25926	0.36003	0.21946
C13	-0.24485	0.33989	0.09478
C14	-0.20951	0.3525	0.11854
C15	-0.29734	0.3463	0.20645

C16	-0.31073	0.36869	0.20433
O17	-0.04219	0.43221	0.72763
H18	-0.08361	0.42965	0.2919
H19	-0.14003	0.38307	0.16804
H20	-0.18489	0.43224	0.48144
H21	-0.24581	0.40981	0.47844
H22	-0.26033	0.31467	-0.03273
H23	-0.19927	0.33639	0.02224
H24	-0.29333	0.39593	0.20356
C25	0.03632	0.5196	0.64224
C26	0.02102	0.54045	0.64259
C27	-0.01502	0.52467	0.63727
C28	0.07414	0.53415	0.61504
O29	0.08683	0.51779	0.75037
N30	0.095	0.56489	0.4331
N31	0.13049	0.57762	0.41584
C32	0.15153	0.60752	0.26875
C33	0.18848	0.61936	0.24876
C34	0.20117	0.59786	0.36549
C35	0.23627	0.6095	0.35355
C36	0.25977	0.64268	0.21124
C37	0.24651	0.66384	0.08728
C38	0.2114	0.65248	0.11227
C39	0.29759	0.65502	0.19667
C40	0.30968	0.63155	0.19426
O41	0.04203	0.57642	0.67598
H42	0.08477	0.57785	0.27996
H43	0.14196	0.62386	0.15921
H44	0.18407	0.5722	0.47622
H45	0.24457	0.59249	0.47102
H46	0.2627	0.68891	-0.04065
H47	0.20204	0.66933	0.01623
H48	0.29148	0.6045	0.19327
C49	1.03041	1.59774	0.4974
C50	0.97169	1.41243	0.89408
H51	0.02738	0.46812	0.67769
H52	-0.02705	0.54037	0.6361
C53	1.06169	1.63261	0.39259
C54	1.05216	1.65923	0.24947
C55	0.94196	1.37661	1.00048
C56	0.9538	1.35171	1.14248
H57	1.01212	1.60099	0.68317
H58	1.01486	1.58477	0.2389
H59	0.98716	1.42611	1.14938
H60	0.99039	1.41043	0.70317
H61	1.07751	1.62761	0.18597
H62	1.07991	1.64452	0.63356
H63	1.03492	1.66317	0.44281

H64	1.0357	1.64899	-0.00317
H65	0.92389	1.3638	0.76025
H66	0.9253	1.38029	1.2079
H67	0.97009	1.36268	1.39491
H68	0.97192	1.34921	0.94889
S69	0.91649	1.30909	1.22908
C70	0.94057	1.28772	1.369
C71	0.91608	1.25145	1.51976
S72	1.09117	1.70057	0.16272
C73	1.06961	1.72447	0.02282
C74	1.09584	1.7602	-0.12794
H75	0.96002	1.30403	1.58454
H76	0.9565	1.28573	1.13831
H77	0.93078	1.2415	1.68155
H78	0.90247	1.23305	1.279
H79	0.89591	1.25145	1.70781
H80	1.05447	1.72772	0.25353
H81	1.04971	1.70904	-0.19269
H82	1.11522	1.7587	-0.316
H83	1.11028	1.77823	0.11281
H84	1.08245	1.77159	-0.28973

Supplementary Table 15 | Fractional atomistic coordinates for Pawley-refined unit cell parameters of TFPB-THz COF (space group *P3*, a = b = 22.6005 Å, c = 3.4540 Å, α = β = 90°, and γ = 120°).

Atom	x	y	z
C1	0.38007	0.73796	0.46353
C2	0.18652	0.61609	0.46688
C3	0.14001	0.54974	0.60268
C4	0.06989	0.52517	0.59968
C5	0.04408	0.56684	0.47364
C6	0.08952	0.63308	0.34484
C7	0.15958	0.65707	0.33514
C8	-0.02975	0.54204	0.47319
N9	-0.0727	0.48092	0.59234
N10	-0.14334	0.45546	0.59108
C11	-0.18663	0.39096	0.72657
C12	-0.26179	0.36198	0.73023
O13	-0.16299	0.35595	0.84129
C14	-0.2909	0.40413	0.73427
H15	0.15688	0.51665	0.72445
H16	0.03602	0.47394	0.70535
H17	0.07062	0.66585	0.24179
H18	0.19164	0.70714	0.21166
H19	-0.0481	0.5753	0.36987
H20	-0.16224	0.48509	0.46973
H21	-0.25864	0.45893	0.74575

C22	0.30922	0.71254	0.46292
H23	0.25216	0.54278	0.46253

Supplementary Table 16 | Fractional atomistic coordinates for Pawley-refined unit cell parameters of DFDM-THz COF (space group *P3*, a = b = 30.4764 Å, c = 3.4231 Å, α = β = 90°, and γ = 120°).

Atom	x	y	z
C1	-0.35019	0.28106	0.28551
C2	-0.29832	0.31665	0.28133
C3	-0.22622	0.40681	0.29107
N4	-0.19102	0.39511	0.42181
N5	-0.1397	0.43225	0.42789
C6	-0.10488	0.42176	0.52047
O7	-0.21233	0.44978	0.18187
C8	-0.05114	0.4617	0.52874
C9	-0.03778	0.51285	0.52618
C10	0.01315	0.55141	0.54203
O11	0.02737	0.60297	0.53557
C12	-0.00462	0.61796	0.72615
H13	-0.27167	0.30326	0.27008
H14	-0.20194	0.35938	0.53394
H15	-0.11525	0.38312	0.58723
H16	-0.06717	0.52264	0.50758
H17	-0.03248	0.58854	0.92272
H18	-0.02512	0.62787	0.51126
H19	0.01945	0.65229	0.89943
C20	0.05114	0.5383	0.52874
C21	0.03778	0.48715	0.52618
C22	-0.01315	0.44859	0.54203
C23	0.10488	0.57824	0.52047
N24	0.1397	0.56775	0.4279
N25	0.19102	0.60489	0.42182
C26	0.22622	0.59319	0.29107
C27	0.28106	0.63125	0.28552
C28	0.29832	0.68335	0.28134
O29	0.21233	0.55022	0.18188
O30	-0.02737	0.39703	0.53557
C31	0.00462	0.38204	0.72615
H32	0.06717	0.47736	0.50758
H33	0.11525	0.61688	0.58723
H34	0.20194	0.64062	0.53394
H35	0.27167	0.69674	0.27009
H36	0.02512	0.37213	0.51126
H37	-0.01945	0.34771	0.89943
H38	0.03248	0.41146	0.92272

Supplementary Methods

General Information

All commercially available chemicals and solvents were purchased from Sigma Aldrich, and used as received without further purification. For all the analytic measurements related to COFs, the COFs were stored in dry and used as-synthesized unless specified.

Nuclear Magnetic Resonance spectroscopy (NMR): ^{1}H NMR and ^{13}C NMR spectra were collected on a Bruker AMX500 (500 MHz) spectrometer. Chemical shifts were reported in parts per million (ppm). Residual solvent peak was used as an internal reference.

High resolution mass spectra (HRMS): All HRMS were recorded on a Finnigan/MAT 95XL-T spectrometer in ESI mode.

Fourier transform infrared (FT-IR): All FT-IR spectra were recorded on a Bruker vertex 80v spectrometer under vacuum.

Solid-state UV-vis absorption spectra: UV-vis spectra were measured using Shimadzu UV-2450 UV-visible spectrophotometer.

Photoluminescence (PL) spectra, lifetime and absolute quantum yield (QY): All PL and lifetimes were recorded on a Horiba Fluorolog-3 spectrofluorometer equipped with a FluoroHub R-928 detector. The PL was measured using excitation wavelength of 365 nm from a Tungsten lamp. Lifetimes were recorded using a 374 nm nanoLED as excitation source. Diluted LUDOX® HS-40 colloidal silica was used for lifetime prompt measurement. PLQY were recorded on the HORIBA Fluorolog-3 Photon Counting Spectrofluorometer System with Quanta-*φ* 6-inch integrating sphere. For PLQY measurement, 15 mg of analyte sample was loaded onto the sample holder to fully cover the bottom.

Note that for solid-state absorption, PL, lifetime and QY of model compounds were measured in as-synthesized polycrystalline form (Section D), except for PL and lifetime of DHzDPr and DHzDAll models, which were measured in single crystal form.

Time-resolved emission spectra (TRES): Time-resolved emission spectra measurements were performed using a 380nm femtosecond laser pulse. The excitation source is a mode-locked Ti:sapphire laser (Chameleon Ultra II, Coherent) working with repetition rate of 80 MHz, pulse duration of 140 fs. The second harmonic generation of 760 nm output from the laser was employed to excite the samples. The laser beam was focused by a 10× objective lens (N.A. = 0.30) with a radius of ~ 0.7 µm. The PL signals were collected in reflection geometry via an inverted microscope (Nikon Eclipse Ti). Emission from the sample were collected with the same objective lens and routed via a bundled optical fiber to a monochromator (Acton, Spectra Pro 2300i) coupled with a photon counting photomultiplier (PMT) (PicoQuant, PMA 182), and the signals were processed by using PicoHarp 300. There was no observed sample damage by the laser during the measurements.

Powder X-ray diffraction (PXRD) measurements: PXRD experiments were performed on Wide-angle X-ray diffraction (XRD) patterns and collected on a Bruker D8 Focus Powder X-ray diffractometer using Cu Kα radiation (40 kV, 40 mA) at room temperature.

Single crystal X-ray diffraction measurements: XRD of single crystals were collected from a Bruker D8 Venture Single Crystal X-ray Diffractometer.

Gas sorption measurements: Gas sorption analysis were performed on Quantachrome Instruments Autosorb-iQ (Boynton Beach, Florida USA) with extra-high pure gases. The samples were activated and outgassed at 120 ºC for 8 h before measurement. The Brunauer-Emmett-Teller (BET) surface area and total pore volume were calculated from the N_2 sorption isotherms at 77 K, and the pore size distribution was calculated based on the N_2 sorption isotherm by using Non-Local Density Functional Theory (NL-DFT, a carbon model containing slit/cylindrical pore) model in the Quantachrome ASiQwin 5.0 software package.

Scanning electron microscopy (SEM): SEM images were taken on a JEOL JSM-6701F Field-Emission microscope. The COFs were dispersed in ethanol and drop-casted onto silica substrates. After drying, the substrates were sputtered with ~9 nm platinum for measurement.

Transmission electron microscopy (TEM): TEM images were taken on an FEI Titan 80-300 S/TEM (Scanning/Transmission Electron Microscope) operated at 200 kV. Similar to SEM measurement, the COF dispersions in ethanol were drop-casted to SPI copper grid (holey carbon, 200-mesh) and dried for TEM measurement.

Elemental analysis: Elemental analysis was performed on Elementar vario MICRO cube for C, H, N and S.

Structural Modelling Method: Molecular modelling and Pawley refinement were conducted using Reflex, a software package for crystal determination from Powder XRD pattern, implemented in BIOVIA Materials Studio modelling version, 2016 (Dassault System).[1] For structural determination of the COFs, the lattice model was optimized using the Materials Studio Forcite molecular dynamics module under ultra-fine, Universal force fields, Ewald summations condition. Then Pawley refinement was performed to optimize the lattice parameters until the R_{wp} value converged. The pseudo-Voigt profile function and Berrar–Baldinozzi function were used for whole profile fitting and asymmetry correction respectively during the refinement process. To further understand the hydrogen bonding environment in the COFs, more accurate models were relaxed and optimized from the previously refined structures using Density Functional Theory (DFT), with the fixed cell shape and cell parameters, i.e. a = b = 29.37 Å, c = 3.44 Å for Tf-DHzDPr, a = b = 29.35 Å, c = 3.43 Å for Tf-DHzDAll. vdW-DF2 method[2] was utilized to take the long-range van der Waals interactions into account, especially for the system with hydrogen bonding. vdW-DF2 calculation was performed to treat the system using Quantum Espresso (QE) package, with a 60 Ry kinetic energy cutoff, 1×1×10 Monkhost-Pack k-mesh and a 10^{-6} Ry convergence threshold.

General Synthetic Procedure for Hydrazides

To a solution of 2,5-dihydroxyterephthalate (1 g, 3.9 mmol) in acetone (70 mL), potassium carbonate (4.5 g, 32.6 mmol) and potassium iodide (80 mg, 0.49 mmol) were added. Then relative iodide (for DHzDM) or bromides (20 mmol) was added into the mixture and the solution was heated at reflux for 2 days. The precipitate was removed by filtration and washed was acetone. The filtrate was evaporated to dryness and the residue was purified by flash column chromatography. The solid obtained was suspended in 50 to 80 mL of ethanol and 5 mL of hydrazine hydrate. The mixture was heat at reflux for 12 h. The mixture was allowed to cool to room temperature and the precipitate was collected by filtration and washed with water and ethanol. The white solid was dried to yield respective desired product.[4, 5]

2,5-Dimethoxyterephthalohydrazide (**DHzDM**): White solid (0.79 g, 80% yield). **^{1}H NMR** (500 MHz, DMSO-*d*$_6$) δ 9.33 (s, 2H), 7.38 (s, 2H), 4.57 (s, 4H), 3.84 (s, 6H). **ESI-HRMS:** Calcd. for [$C_{10}H_{14}N_4O_4$-H] 253.0942, found 253.0941.

2,5-Dipropoxyterephthalohydrazide (**DHzDPr**): White crystal (1.03 g, 85% yield). **^{1}H NMR** (500 MHz, DMSO-*d*$_6$) δ 9.21 (s, 2H), 7.38 (s, 2H), 4.58 (s, 4H), 4.03 (t, *J* = 6.5 Hz, 4H), 1.83 – 1.69 (m, 4H), 0.98 (t, *J* = 7.4 Hz, 6H). **^{13}C NMR** (126 MHz, DMSO-*d*$_6$) δ 163.82, 149.71, 125.02, 114.72, 70.62, 21.90, 10.37. **ESI-HRMS:** Calcd. for [$C_{14}H_{22}N_4O_4$-H] 309.1568, found 309.1570.

2,5-Bis(allyloxy)terephthalohydrazide (**DHzDAll**): White crystal (0.97 g, 82% yield). **^{1}H NMR** (500 MHz, DMSO-*d*$_6$) δ 9.27 (t, *J* = 3.4 Hz, 2H), 7.34 (s, 2H), 6.05 (ddt, *J* = 17.3, 10.4, 5.1 Hz, 2H), 5.39 (dd, *J* = 17.3, 1.7 Hz, 2H), 5.28 (dd, *J* = 10.6, 1.5 Hz, 2H), 4.66 (d, *J* = 5.1 Hz, 4H), 4.56 (d, *J* = 4.3 Hz, 4H). **^{13}C NMR** (126 MHz, DMSO-*d*$_6$) δ 163.79, 149.30, 133.33, 125.32, 117.72, 114.97, 69.56. **ESI-HRMS:** Calcd. for [$C_{14}H_{18}N_4O_4$-H] 305.1255, found 305.1254.

2,5-Bis(3-(ethylthio)propoxy)terephthalohydrazide (**DHzDS**): White solid (1.2 g, 72% yield). **^{1}H NMR** (500 MHz, DMSO-*d*$_6$) δ 9.22 (s, 2H), 7.34 (s, 2H), 4.55 (s, 4H), 4.13 (t, *J*= 6.2 Hz, 4H), 2.65 (t, *J* = 7.2 Hz, 4H), 2.55 – 2.51 (m, 4H), 1.99 (p, *J* = 6.5 Hz, 4H), 1.19 (t, *J* = 7.4 Hz, 6H). **ESI-HRMS:** Calcd. for [$C_{18}H_{30}N_4O_4S_2$-H] 429.1636, found 429.1639.

Benzene-1,3,5-tricarbohydrazide (**THz**): To a suspension of trimethyl benzene-1,3,5-tricarboxylate (5 g, 20 mmol) in ethanol/toluene (120 mL, v/v= 1:1), hydrazine hydrate (10 mL) was added. The mixture was heated to reflux for 15 h. The mixture was hot filtered and the solid was washed with extensive ethanol. Then the solid was vacuumed to dryness to obtain a white solid (4.1 g, 81% yield). **^{1}H NMR** (500 MHz, DMSO-*d*$_6$) δ 9.83 (s, 2H), 8.32 (s, 2H), 4.57 (s, 4H). **ESI-HRMS:** Calcd. for [$C_9H_{12}N_6O_3$-H] 251.0898, found 251.0891.

General Synthetic Procedure of Tuneable White-emissive COFs

2,5-Disubstituted terephthalohydrazide (0.075 mmol) or benzene-1,3,5-tricarbohydrazide (0.05 mmol), was mixed with relative benzaldehydes in a 10 mL Schlenk tube (15 mm × 80 mm). A mixture of 1,4-dioxane and mesitylene (0.8 mL ~ 2 mL, 1:3) was added into the tube and sonicated for 10 minutes. The mixture was then added with acetic acid (50~200 μL, 6 M), flash frozen at 77 K, and degassed under freeze-pump-thaw for three cycles. The tube was sealed and heated at 120 °C for three days. The solid obtained was exchanged with anhydrous THF (5 mL) for 10 times and dried at 100 °C under vacuum for 8 hours to yield corresponding COFs.

Tf-DHzDM COF: white solid (19.6 mg, 80%). FT-IR (KBr, cm^{-1}): 3433, 3274, 2974, 2939, 2846, 1668, 1622, 1537, 1487, 1460, 1398, 1303, 1228, 1167, 1081, 1027, 968, 893, 760, 580. Anal. Calcd. for $(C_{24}H_{21}N_6O_6)_n$: C 58.89; H 4.32; N 17.17; O 19.61; found: C 48.02; H 6.13; N 12.42.

Tf-DHzDPr COF: white solid (24.7 mg, 86%). FT-IR (KBr, cm^{-1}): 3450, 3272, 2968, 2927, 2877, 1672, 1620, 1533, 1487, 1415, 1388, 1220, 997, 814, 577. Anal. Calcd. for $(C_{30}H_{33}N_6O_6)_n$: C 62.82; H 5.8; N 14.65; O 16.73; found: C 55.81; H 5.64; N 12.14.

Tf-DHzDAll COF: Beige solid (25.1 mg, 88%). FT-IR (KBr, cm^{-1}): 3435, 3261, 2918, 1664, 1641, 1535, 1488, 1412, 1224, 1080, 999, 949, 814, 771, 579. Anal. Calcd. for $(C_{30}H_{33}N_6O_6)_n$: C 63.49; H 4.79; N 14.81; O 16.91; found: C 57.21; H 5.34; N 12.73.

TFPB-DHzDM COF: Beige solid (33.1 mg, 92%). FT-IR (KBr, cm^{-1}): 3413, 3272, 2935, 2846, 1658, 1602, 1537, 1487, 1463, 1396, 1301, 1209, 1171, 1026, 825, 756, 571, 538.Anal. Calcd. for $(C_{42}H_{33}N_6O_6)_n$: C 70.28; H 4.63; N 11.71; O 13.37; found: C 64.77; H 4.95; N 11.06.

TFPB-DHzDPr COF: Pale green solid (35.5 mg, 89%). FT-IR (KBr, cm^{-1}): 3440, 3377, 3280, 3055, 3028, 2962, 2931, 2875, 1666, 1602, 1535, 1488, 1413, 1386, 1209, 1062, 1002, 976, 825, 800, 768, 577, 536. Anal. Calcd. for $(C_{48}H_{45}N_6O_6)_n$: C 71.89; H 5.66; N 10.48; O 11.97; found: C 66.92; H 5.64; N 9.72.

TFPB-DHzDAll COF: Beige solid (37.9 mg, 95%). FT-IR (KBr, cm^{-1}): 3450, 3280, 2916, 2848, 1643, 1602, 1479, 1407, 1232, 993, 926, 824, 802, 532. Anal. Calcd. for $(C_{48}H_{39}N_6O_6)_n$: C 72.44; H 4.94; N 10.56; O 12.06; found: C 67.81; H 5.04; N 9.86.

TFPB-DHzDS COF: Pale green solid (40.8 mg, 83%). FT-IR (KBr, cm^{-1}): 3460, 3280, 2960, 2921, 2869, 1668, 1602, 1537, 1487, 1413, 1380, 1209, 1014, 825, 800, 575. Anal. Calcd. for $(C_{54}H_{57}N_6O_6S_3)_n$: C 66.03; H 5.85; N 8.56; S 9.79; O 9.77; found: C 62.5; H 5.86; N 7.5; S 7.99.

TFPB-THz COF: Beige solid (27.1 mg, 92%). FT-IR (KBr, cm^{-1}): 3421, 3207, 3039, 2914, 2848, 1664, 1600, 1544, 1508, 1440, 1359, 1261, 1068, 825, 733. Anal. Calcd. for $(C_{36}H_{24}N_6O_3)_n$: C 73.46; H 4.11; N 14.28; O 8.15; found: C 66.34; H 5.05; N 11.48.

DFDM-THz COF: Yellow solid (23.4 mg, 96%). FT-IR (KBr, cm^{-1}): 3440, 3290, 3191, 3055, 2945, 2848, 1677, 1618, 1550, 1409, 1259, 1213, 1037, 735, 681, 615. Anal. Calcd. for $(C_{24}H_{21}N_6O_6)_n$: C 58.89; H 4.32; N 17.17; O 19.61; found: C 51.31; H 5.01; N 16.12.

General Synthetic Procedure of Model Compounds

DHzDR Models and **THz Model:** Relative hydrazide (0.20 mmol) was dispersed in methanol (10 mL). The mixture was added with benzaldehyde (10 eq) and heated at refluxed for 2 days under argon atmosphere. The precipitate was filtered, washed with methanol and dried at 70 °C under vacuum for 8 hours to yield respective desired products.

DHzDM Model: White solid (71 mg, 82% yield). **^{1}H NMR** (500 MHz, DMSO-*d*$_6$) (major isomer) δ 11.55 (s, 2H), 8.39 (s, 2H), 7.74 (d, *J* = 7.6 Hz, 4H), 7.47 (d, *J* = 6.4 Hz, 6H), 7.43 (s, 2H), 3.89 (d, *J* = 18.8 Hz, 6H). **ESI-HRMS:** Calcd. for [$C_{24}H_{22}N_4O_4$-H] 429.1568, found 429.1570.

DHzDPr Model: White solid (84 mg, 86% yield). **^{1}H NMR** (500 MHz, DMSO-*d*$_6$) (major isomer) δ 11.54 (s, 2H), 8.30 (s, 2H), 7.73 (d, *J* = 6.3 Hz, 4H), 7.52 – 7.42 (m, 6H), 7.38 (s, 2H), 4.05 (dt, *J* = 17.5, 6.5 Hz, 4H), 1.81 – 1.73 (m, 4H), 0.98 (t, *J* = 7.4 Hz, 6H). **ESI-HRMS:** Calcd. for [$C_{28}H_{30}N_4O_4$-H] 485.2194, found 485.2197.

DHzDAll Model: White solid (74 mg, 77% yield). **^{1}H NMR** (500 MHz, DMSO-*d*$_6$) (major isomer) δ 11.58 (s, 2H), 8.32 (s, 2H), 7.74 (d, *J* = 7.7 Hz, 4H), 7.47 (q, *J* = 5.9 Hz, 6H), 7.39 (s, 2H), 6.14 – 6.01 (m, 2H), 5.41 (dd, *J* = 17.2, 11.8 Hz, 2H), 5.29 – 5.23 (m, 2H), 4.69 (dd, *J* = 18.1, 4.6 Hz, 4H). **^{13}C NMR** (126 MHz, DMSO-*d*$_6$) (major isomer) δ 161.41, 149.42, 147.54, 134.18, 133.38, 130.16, 128.86, 127.11, 117.51, 116.76, 115.01, 69.70. **ESI-HRMS:** Calcd. for [$C_{28}H_{26}N_4O_4$-H] 481.1881, found 481.1887.

DHzDS Model: White solid (97 mg, 80% yield). **^{1}H NMR** (500 MHz, DMSO-*d*$_6$) (major isomer) δ 11.55 (s, 2H), 8.31 (s, 2H), 7.72 (d, *J* = 7.8 Hz, 4H), 7.52 – 7.44 (m, 6H), 7.38 (s, 2H), 4.20 – 4.12 (m, 4H), 2.67 – 2.62 (m, 4H), 2.43 (dd, *J* = 7.5, 4.2 Hz, 4H), 2.00 (dd, *J* = 13.6, 6.6 Hz, 4H), 1.13 – 1.06 (m, 6H). **ESI-HRMS:** Calcd. for [$C_{32}H_{38}N_4O_4S_2$-H] 605.2262, found 605.2256.

THz Model: White solid (87 mg, 84% yield). **^{1}H NMR** (500 MHz, DMSO-*d*$_6$) (major isomer) δ 12.19 (s, 1H), 8.66 (s, 1H), 8.53 (s, 1H), 7.78 (d, *J* = 6.0 Hz, 2H), 7.49 (d, *J* = 6.6 Hz, 3H). **^{13}C NMR** (126 MHz, DMSO-*d*$_6$) (major isomer) δ 161.97, 148.56, 134.13, 130.23, 129.81, 128.83, 127.19. **ESI-HRMS:** Calcd. for [$C_{30}H_{24}N_6O_3$-H] 515.1837, found 515.1840.

General Synthetic Procedure of Multi-Component COFs

Various mole ratios of 2,5-disubstituted terephthalohydrazides (0.075 mmol, 2:1, 1:1 or 1:2), was mixed with relative benzaldehydes (0.05 mmol) in a 10 mL Schlenk tube (15 mm × 80 mm). A mixture of 1,4-dioxane and mesitylene (0.8 mL ~ 2 mL, 1:3) was added into the tube and sonicated for 10 minutes. The mixture was then added with acetic acid (50~200 μL, 6 M), flash frozen at 77 K, and degassed under freeze-pump-thaw for three cycles. The tube was sealed and heated at 120 °C for three days. The solid obtained was exchanged with anhydrous THF (5 mL) for several times and dried at 100 °C under vacuum for 8 hours to yield corresponding COFs.

Tf-DHz2DAll1DPr COF: Beige solid (25.6 mg, 90%). FT-IR (KBr, cm^{-1}): 3454, 3274, 2966, 2873, 1674, 1620, 1535, 1487, 1413, 1226, 1078, 999, 945, 814, 771, 685, 577. Anal. Calcd. for $(C_{30}H_{29}N_6O_6)_n$: C 63.26; H 5.13; N 14.75; O 16.85; found: C 56.64; H 5.15; N 12.58.

Tf-DHz1DAll1DPr COF: Beige solid (25.3 mg, 89%). FT-IR (KBr, cm^{-1}): 3454, 3271, 2966, 2933, 2877, 1668, 1616, 1535, 1488, 1413, 1384, 1224, 1078, 999, 949, 814, 771, 687, 577. Anal. Calcd. for $(C_{30}H_{30}N_6O_6)_n$: C 63.15; H 5.30; N 14.73; O 16.82; found: C 56.47; H 5.40; N 12.28.

Tf-DHz1DAll2DPr COF: Beige solid (25.6 mg, 92%). FT-IR (KBr, cm^{-1}): 3450, 3271, 2966, 2933, 2879, 1666, 1618, 1535, 1488, 1415, 1386, 1226, 1078, 999, 949, 814, 771, 689, 577. Anal. Calcd. for $(C_{30}H_{31}N_6O_6)_n$: C 63.04; H 5.47; N 14.70; O 16.79; found: C 56.51 ; H 5.36; N 12.50.

TFPB-DHz2DAll1DPr COF: Beige solid (34 mg, 85%). FT-IR (KBr, cm^{-1}): 3431, 3274, 2966, 2933, 2875, 1662, 1604, 1542, 1413, 1213, 1066, 1000, 932, 824, 802, 575, 536. Anal. Calcd. for $(C_{48}H_{41}N_6O_6)_n$: C 72.26; H 5.18; N 10.53; O 12.03; found: C 67.84; H 5.16; N 9.46.

TFPB-DHz1DAll2DPr COF: Beige solid (34.6 mg, 87%). FT-IR (KBr, cm^{-1}): 3431, 3274, 2966, 2933, 2875, 1664, 1604, 1542, 1413, 1386, 1213, 1066, 1000, 932, 824, 802, 575, 536. Anal. Calcd. for $(C_{48}H_{43}N_6O_6)_n$: C 72.07; H 5.42; N 10.51; O 12.00; found: C 67.98; H 5.11; N 9.87.

TFPB-DHz2DAll1DS COF: Beige solid (36.9 mg, 86%). FT-IR (KBr, cm^{-1}): 3435, 3278, 2952, 2848, 1666, 1600, 1537, 1412, 1209, 1012, 824, 798, 577, 534. Anal. Calcd. for $(C_{50}H_{45}N_6O_6S)_n$: C 69.99; H 5.29; N 9.80; O 11.19; S 3.74; found: C 66.53; H 5.44; N 8.48; S 3.43.

TFPB-DHz1DAll2DS COF: Beige solid (40.5 mg, 88%). FT-IR (KBr, cm^{-1}): 3435, 3282, 2956, 2921, 2848, 1666, 1600, 1537, 1412, 1213, 1012, 824, 802, 575, 536. Anal. Calcd. for $(C_{52}H_{51}N_6O_6S_2)_n$: C 67.88; H 5.59; N 9.13; O 10.43; S 6.97; found: C 63.54; H 5.45; N 8.03; S 5.54.

Supplementary Notes

Supplementary Note 1

In this work, PBE exchange functional was used in quantum espresso.[11]

In the code:

“vdw-df2” exchange correlation functional = "sla+pw+rw86+vdw2" = vdW-DF2

Exchange: sla = slater;

Correlation: pw = Perdew Wang

Gradient correction on exchange: rw86 = revised PW86

Van der Waals functional: vdW

The details of the vdw-DF2 functional are given in Supplementary Reference 12.

Supplementary Discussion

Mechanism study of ESIPS in dual-emissive COFs

The dual-emissive Tf-DHzDAll COF is structurally quite similar to the single-emissive Tf-DHzDPr COF, except that the side-chain substituent on the Tf-DHzDAll COF is an allyl group instead of a propyl group. Here, we discuss in detail the evidence that the dual emission is related to the presence of significant interlayer hydrogen bonding in Tf-DHzDAll COF. Our DFT studies using the vdW-DF2 functionals[2] revealed an interlayer hydrogen bonding distance of 2.40 Å and an intralayer hydrogen bonding distance of 2.33 Å for dual-emissive Tf-DHzDAll COF and an interlayer hydrogen bonding distance of 3.00 Å and an intralayer hydrogen bonding distance of 2.03 Å for single-emissive Tf-DHzDPr COF (Fig. 1d and Supplementary Fig. 31 and 32). These distances indicate clearly that interlayer hydrogen bonding is an important distinguishing feature of Tf-DHzDAll COF compared to Tf-DHzDPr COF. Furthermore, time-dependent DFT calculations on single molecule fragments extracted from the COF compounds indicate that changing the propyl group to the allyl group does not change the emission peak significantly in the gas phase molecules (see Supplementary Table 7). These distances suggest that the competition between intra- and interlayer hydrogen bonding can result in different dominant relaxation pathways during de-excitation. Electrospray ionization mass spectrometry analysis of the model compounds shows that the negatively charged deprotonated form is the major species present, suggesting that the O=C-N-H proton is acidic. This implies the possibility of a proton shift between adjacent COF layers in the excited state. Therefore, the overall process of dual emission is proposed as shown in the energy diagram (Supplementary Figure 33a). Tf-DHzDAll adopts conformer C1 with a ground-state structure closed to the simulated result. After photoexcitation, Tf-DHzDAll first converts to C1* with a similar electronic structure to the ground state and then tautomerizes to C2* via proton shift, which has a different intra- and interlayer hydrogen bonding environment. Finally, both C1* and C2* undergo radiative de-excitation to give the observed dual emission and return to the ground state.

Time-resolved emission spectra confirm the existence of two distinct emissive species, C1* and C2* (Supplementary Fig. 30b) with different lifetimes. The minor emission band at approximately 484 nm, with a smaller Stokes shift, is assigned to the conformer (C1*), which has a similar electronic structure to ground state according to reported ESIPT theory. Meanwhile, the major emission band at approximately 545 nm is attributed to the ESIPS product (C2*), due to the non-radiative energy dissipation from conformational change. The C2* isomer exhibits a longer PL lifetime than the C1* form. This observation provides further evidence of the existence of a second conformer via proton shift. From ESIPT theory, we know that a COF-triggered ESIPS tautomer has a longer recombination lifetime.[8,9] To further confirm the dynamic equilibrium process of ESIPS, temperature-dependent PL experiments were also performed (Supplementary Fig. 33c). With increasing temperature, the ratio of the populations of C1* and C2* decreases, suggesting that the equilibrium shifts to the right. When the solid is cooled, the tautomerization equilibrium shifts to the left, and the population of the C1* isomer increases. This agrees well with the endergonic ESIPT process, in which thermal energy can shift the equilibrium to the redshift isomer.[10] A further proof of the dynamic equilibrium

involving proton-shift comes from the sensitivity of Tf-DHzDAll COF to humidity, where exposure to water vapour reduces the intensity of the redshifted peak, and drying the COF restores its intensity (Supplementary Fig. 30). The presence of water disturbs the equilibrium of intra- and interlayer hydrogen bonds in the COF and affects the proton shift, leading to an increased population of the blueshift PL emission.

Absence of Intramolecular Proton Transfer in DHzDAll and DHzDPr Model Compounds

We have confirmed that the ESIPT (excited-state intramolecular proton transfer) pathway, based on tautomerization between hydrazide and hydrazonate (Supplementary Fig. 34), is not favoured in our COF system, as concluded from our DFT and TDDFT (time-dependent density functional theory) calculation results for the simulated molecular fragments (treated as gas phase single molecules). The geometries after artificial proton transfer were not stable both in their ground state and excited state and returned back to their respective initial structures after optimization (Supplementary Fig. 35). We first constructed the hydrazonate form (after-proton-transfer structures) by enforcing the formation of O-H bond artificially, and then fully relaxed the geometries by using B3LYP hybrid exchange correlation functional with 6-31++g** basis sets. The ground state geometries were optimized within the framework of DFT while the excited state geometry optimization was performed using TDDFT (as implemented both in Gaussian 16[6] and NWChem[7], without the pre-optimization in ground state).

We also report the simulated absorption and emission peaks for the single molecule DHzDAll and DHzTPr models, based on Kasha's rule using TDDFT with CAM-B3LYP and B3LYP functionals, with 6-31++g** basis sets. We find that the change from allyl group and propyl group does not affect the emission energy significantly (Supplementary Table 7). This provides further evidence that interlayer hydrogen bonding is required to explain the extra lower energy emission peak found in the COF structures.

Supplementary References

1. Dassault Systèmes BIOVIA, Materials Studio, Version 2016, San Diego: Dassault Systèmes (2017).
2. Dion, M., Rydberg, H., Schröder, E., Langreth, D. C. & Lundqvist, B. I. Van der Waals Density Functional for General Geometries. *Phys. Rev. Lett.* **92,** 246401 (2004).
3. Paolo, G. *et al.* QUANTUM ESPRESSO: a modular and open-source software project for quantum simulations of materials. *J. Phys. Condens. Matter* **21,** 395502 (2009).
4. Uribe-Romo, F. J., Doonan, C. J., Furukawa, H., Oisaki, K. & Yaghi, O. M. Crystalline Covalent Organic Frameworks with Hydrazone Linkages. *J. Am. Chem. Soc.* **133,** 11478-11481 (2011).
5. Ding, S.-Y. *et al.* Thioether-Based Fluorescent Covalent Organic Framework for Selective Detection and Facile Removal of Mercury(II). *J. Am. Chem. Soc.* **138,** 3031-3037 (2016).
6. Frisch, M. J., *et al.* Gaussian 16. 2016: Wallingford, CT.
7. Valiev, M. *et al.* NWChem: A comprehensive and scalable open-source solution for large scale molecular simulations. *Comput. Phys. Commun*. **181,** 1477-1489 (2010).
8. Shynkar, V. V. *et al.* Picosecond Time-Resolved Fluorescence Studies Are Consistent with Reversible Excited-State Intramolecular Proton Transfer in 4‘-(Dialkylamino)-3-hydroxyflavones. *J. Phys. Chem. A* **107,** 9522-9529 (2003).
9. Zhao, J., Ji, S., Chen, Y., Guo, H. & Yang, P. Excited state intramolecular proton transfer (ESIPT): from principal photophysics to the development of new chromophores and applications in fluorescent molecular probes and luminescent materials. *Phys. Chem. Chem. Phys.* **14,** 8803-8817 (2012).
10. Tang, K.-C. *et al.* Fine Tuning the Energetics of Excited-State Intramolecular Proton Transfer (ESIPT): White Light Generation in A Single ESIPT System. *J. Am. Chem. Soc.* **133,** 17738-17745 (2011).
11. Perdew, J.P., Burke, K. & Ernzerhof, M. Generalized Gradient Approximation Made Simple. *Phys. Rev. Lett.* **77**, 3865-3868 (1996).
12. Lee, K., Murray, E. D., Kong, L., Ludqvist, B. I. & Langreth D. C. Higher-accuracy van der Waals density functional. *Phys. Rev. B* **82**, 081101 (2010).
